# Synthesizing Configurable Biochemical Implementation of Linear Systems from Their Transfer Function Specifications

**DOI:** 10.1371/journal.pone.0137442

**Published:** 2015-09-09

**Authors:** Tai-Yin Chiu, Hui-Ju K. Chiang, Ruei-Yang Huang, Jie-Hong R. Jiang, François Fages

**Affiliations:** 1 Department of Physics, National Taiwan University, Taipei, Taiwan; 2 Graduate Institute of Electronics Engineering, National Taiwan University, Taipei, Taiwan; 3 EPI Lifeware, Inria Paris-Rocquencourt, Rocquencourt, France; 4 Department of Electrical Engineering, National Taiwan University, Taipei, Taiwan; Virginia Tech, UNITED STATES

## Abstract

The ability to engineer synthetic systems in the biochemical context is constantly being improved and has a profound societal impact. Linear system design is one of the most pervasive methods applied in control tasks, and its biochemical realization has been proposed by Oishi and Klavins and advanced further in recent years. However, several technical issues remain unsolved. Specifically, the design process is not fully automated from specification at the transfer function level, systems once designed often lack dynamic adaptivity to environmental changes, matching rate constants of reactions is not always possible, and implementation may be approximative and greatly deviate from the specifications. Building upon the work of Oishi and Klavins, this paper overcomes these issues by introducing a design flow that transforms a transfer-function specification of a linear system into a set of chemical reactions, whose input-output response precisely conforms to the specification. This system is implementable using the DNA strand displacement technique. The underlying configurability is embedded into primitive components and template modules, and thus the entire system is adaptive. Simulation of DNA strand displacement implementation confirmed the feasibility and superiority of the proposed synthesis flow.

## Introduction

Advancements in synthetic biology have resulted in the development of biochemical systems of increasing complexity that are capable of using living cells as well as cell-free systems. Synthetic biology holds promise for biotechnology, biomedicine, bio-environmental, bioenergy, and other applications. Many computation and control design examples have been demonstrated either *in vivo* or *in vitro*. For instance, oscillators [1], toggle switches [2], logic gates [3], band pass filters [4], and analog circuits [5] have been designed and implemented in living cells, while digital circuits [6], neural networks [7], and switchable memories [8] have been demonstrated in cell-free systems. These developments show a clear growing trend in design complexity. Indeed, the more sophisticated systems we can build, the better skills we will have to comprehend biology and to build even more sophisticated systems.

The increasing system complexity renders the necessity of design automation tools. Constructing biochemical systems bottom-up using pre-characterized parts in synthetic biology is analogous to designing electronic systems using pre-designed standard modules. While electronic design automation (EDA) has largely enabled system design under exponential capacity growth over the past five decades thanks to the driving force of Moore’s law, design automation in synthetic biology may play a similar key role in the construction of complex biochemical systems [9]. Computer-aided modeling, simulation, synthesis, and verification are crucial because a biochemical design is often intended to function in a biological context that is often too complex to be fully characterized.

Depending on its target application, a system is often designed for a specific application rather than as a general purpose computing machine. In control applications, linear systems are pervasive due to their simplicity of design and analysis. Any linear control system can be realized with three primitive components: integration, gain, and summation. Realizing linear control with biochemical reactions has been proposed by Oishi and Klavins [10], who showed that three types of chemical reactions (catalysis, degradation, and annihilation) are sufficient to implement the three primitive components. In principle, any polynomial ordinary differential equation can be approximated by chemical reaction networks (CRNs) [11]. Therefore, any linear control system can be built using CRNs.

To realize CRNs, nucleic acids have been exploited as a universal tool for biomolecular computation [12]. By appropriately changing their nucleotide sequences, the interaction between nucleic acids can be precisely controlled and programmed *in vitro*. Specifically, toehold-mediated or enzyme-free DNA strand displacement (DSD) is a promising approach to perform biological computations. Although the DSD mechanism has been studied since the 1970s [13–17], until recently it was systematically used to build a molecular machine made of DNA and RNA [18]. The DSD method is attractive for several reasons. First, the kinetics of DSD devices can be engineered by controlling toehold binding rates, which may range from 1 to 6 × 10^6^ M^−1^ s^−1^ [19–21]. Second, composability [22] can be achieved. In typical implementation schemes, single-stranded DNAs play the role of signals and double-stranded DNAs act as gates, and they work together with the mechanism of toehold-mediated DNA displacement [19]. DNA signals of different domain lengths [23–25] have been proposed. For example, gates can be composed to realize complex systems using a 2-domain signal architecture [25]. Third, the DSD mechanism works autonomously [26] as long as the DNA or RNA fuels are supplied. With these advantages, many biomolecular devices, e.g., [27–29], have been designed using this powerful mechanism. The DSD technology allows computation and interfacing with molecular components in living organisms [30] and shows potential in bio-sensing and control, biomedicine, and other applications.

Despite the advancement of biochemical implementation of linear systems, four challenges remain to be solved. First, the construction of Oishi and Klavins’s system requires that the rate constants of the underlying reactions be carefully matched to achieve the intended integration, gain, and summation functions. This requirement imposes substantial practicality restrictions because, in reality, the reaction rates of available reactions can be limited. Thus, not all gain and summation blocks can be realized. Second, once a system is constructed, its function is fixed and cannot be changed without redesign. However, this fixed functionality can be inadequate for a system reacting to its biochemical environment, which is intrinsically stochastic and often full of uncertainty. Thus, designing systems with dynamic adaptation capabilities is crucial, especially in the biochemical context. Third, the CRN implementations of the gain and summation components are approximative. In essence, the transfer functions of the gain and summation components contain extra poles rather than just the ideal scalar and summation. When a complex system is built from these components, these additional poles might cause system behavior that deviates from its specification and can even lead to unwanted instability. Fourth, an automated flow of the synthesis of CRN from the transfer function specification of a linear system is lacking. Although a linear system can be compiled into a CRN with a direct block-by-block conversion from a block diagram, the block diagram may not be available in the first place and the CRN implementation may only approximate the specification. Specifying a linear system using transfer functions can be more natural than using block diagrams. However, converting a transfer function into a block diagram suitable for CRN realization can be a nontrivial optimization task.

**Fig 1:**
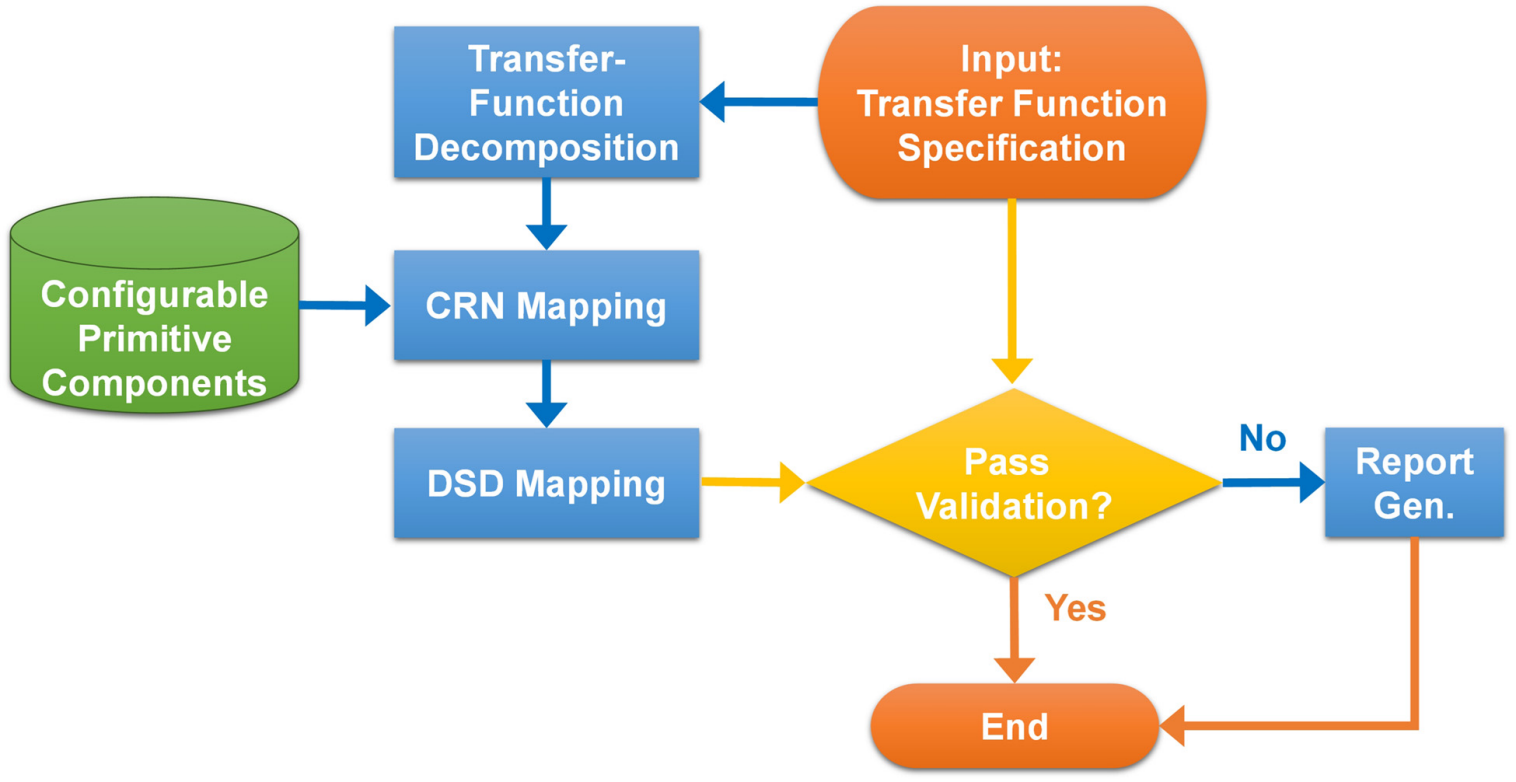
Automated flow of linear system synthesis.

In this paper, we tackle the above challenges. To address the first two challenges, we devise a mechanism to make linear control systems configurable by adding auxiliary species to CRN as control knobs. The concentrations of the auxiliary species can be adjusted not only to compensate for reaction rate mismatch but also to reconfigure a control system to dynamically adapt to environmental changes. Hence, implementing linear control systems in biochemistry can be made more practical. To resolve the last two challenges, we apply parallel decomposition on a given transfer function and express it as a summation of elementary modules, and propose a CRN solution that achieves the exact implementation of the elementary modules. The CRN implementation of a transfer function can be further mapped and realized using the DSD method. The proposed method lends itself to an automated design flow (as depicted in Fig 1) where the linear system to be synthesized is specified by a transfer function, which is decomposed for CRN implementation and further mapped into the DSD reactions. Through simulation using Visual DSD [31, 32], we show the feasibility and superiority of our proposed design automation flow for synthesizing a specified linear system into DSD reactions.

## Methods and Results

Although synthetic biology shows promising potential in biotechnology applications, it poses grand challenges to complex system design. A typical reactive system may involve sensing, actuation, information processing, and other tasks, while a control unit is one of the fundamental ingredients that constitute a reactive system. In this work, we address the design automation problem of linear control systems that are to be realized by biochemical reactions.

Any linear time-invariant (LTI) system can be fully specified by means of a transfer function, which corresponds to the Laplace transform of the impulse response of the system, with all initial conditions set to zero. In essence, once the impulse response of a linear system is known, the output *y*(*t*) of the system with any input *u*(*t*) can be characterized using the transfer function. However, for a multiple-input multiple-output (MIMO) linear system, the principle of superposition suggest that the total effect on any output due to all the inputs acting simultaneously is obtained by summing up the output effects due to the individual inputs acting alone. Consequently any MIMO system can be specified by a matrix of transfer functions. For simplicity, in this paper we focus on single-input single-output (SISO) systems without loss of generality.

### Configurable Primitive Components

A transfer function can be realized by building a network of primitive components consisting of integration, gain, and summation blocks; therefore, implementing an LTI system in biochemical systems amounts to realizing these three basic components with chemical reactions [10]. However, the CRN realizations of these components proposed by Oishi and Klavins [10] are deficient in that rate constants have to be carefully matched and the designed control system cannot be reconfigured. These deficiencies impose a practicality issue that restricts system realization and a flexibility issue that prevents dynamic system adaptation. We can overcome these issues by introducing configurable primitive components as discussed below.

Following Oishi and Klavins [10], we represent a real signal *x* by the difference (*x* ^+^ − *x* ^−^) between the concentrations of two complementary molecular species *x* ^+^ and *x* ^−^. In this paper, we do not distinguish notationally between a species and its concentration. Moreover, we abbreviate a pair of chemical reactions of complementary species

$$\begin{array}{ccc} & & x^{+}+\cdots \;\overset{r^{+}}{\to }\;y^{-}+\cdots ,\\ & & x^{-}+\cdots \;\overset{r^{-}}{\to }\;y^{+}+\cdots , \end{array}$$

with rate constants *r* ^+^ and *r* ^−^, to

$$\begin{array}{c} x^{\pm }+\cdots \;\overset{r^{\pm }}{\to }\;y^{\mp }+\cdots , \end{array}$$

where the plus and minus signs in the superscripts ± and ∓ are ordered.

Table 1 shows the chemical reactions and transfer functions of the proposed configurable primitive components, which we will elaborate on in the following sections.

**Table 1:** Three primitive components, their chemical reactions, and their transfer functions.

Type	Reactions	Transfer Function
Integration	$\left\{ \begin{array}{c} x^{\pm }+u^{\pm }\overset{r_{1}^{\pm }}{\to }x^{\pm }+u^{\pm }+y^{\pm }\\ y^{+}+y^{-}\overset{r_{\text{int}}}{\to }\emptyset \end{array}\right.$	$\begin{array}{c} Y(s)=\frac{k}{s}U(s),\\ k\equiv r_{1}^{+}x^{+}=r_{1}^{-}x^{-} \end{array}$
Gain & Summation	$\left\{ \begin{array}{c} x_{i}^{\pm }+u_{i}^{\pm }\overset{r_{i}^{\pm }}{\to }x_{i}^{\pm }+u_{i}^{\pm }+y^{\pm },i=1,\ldots ,n\\ z^{\pm }+y^{\pm }\overset{r_{0}^{\pm }}{\to }z^{\pm }\\ y^{+}+y^{-}\overset{r_{\text{gs}}}{\to }\emptyset \end{array}\right.$	$\begin{array}{c} Y(s)=\sum_{i=1}^{n}\frac{k_{i}}{s+k_{0}}U_{i}(s),\\ \left\{ \begin{array}{c} k_{i}\equiv r_{i}^{+}x_{i}^{+}=r_{i}^{-}x_{i}^{-},i=1,\ldots ,n\\ k_{0}\equiv r_{0}^{+}z^{+}=r_{0}^{-}z^{-} \end{array}\right. \end{array}$

#### Integration Block

An integration block takes a signal *u*(*t*) as input, and produces a signal $y(t)=k\int_{0}^{t}u(\tau )\text{d}\tau +y(0)$, for some constant *k* ∈ ℝ, as output. The chemical realization of an integration block for *k* ≥ 0 consists of a pair of catalytic reactions

$$\begin{array}{c} x^{\pm }+u^{\pm }\overset{r_{1}^{\pm }}{\to }x^{\pm }+u^{\pm }+y^{\pm } \end{array}$$

and an annihilation reaction

$$\begin{array}{c} y^{+}+y^{-}\overset{r_{\text{int}}}{\to }\emptyset , \end{array}$$

where $r_{1}^{+},r_{1}^{-},r_{\text{int}}$ are the rate constants. The reactions differ from those of Oishi and Klavins [10] in that the auxiliary species *x* ^±^ are newly added to the catalytic reactions. Both the auxiliary species *x* ^±^ and the input species *u* ^±^ serve as catalysts.

With the definition that $r_{1}^{+}x^{+}=r_{1}^{-}x^{-}\equiv k$, the signal *y* is exactly the integration of signal *u* as described by the ordinary differential equation

$$\begin{array}{c} \dot{y}={\dot{y}}^{+}-{\dot{y}}^{-}=r_{1}^{+}x^{+}u^{+}-r_{1}^{-}x^{-}u^{-}=ku, \end{array}$$

for ${\dot{y}}^{\pm }=r_{1}^{\pm }x^{\pm }u^{\pm }-r_{\text{int}}y^{+}y^{-}$. Taking the Laplace transform of the above equation, we obtain the transfer function $\frac{Y}{U}=\frac{k}{s}$.

Because the concentrations of *x* ^+^ and *x* ^−^ can be controlled externally, in theory it is always possible to design a reaction network to meet any required *k*. For *k* < 0, the reactions are the same except that the catalytic reactions should be modified by reversing the complementary species of the output signal *y* as

$$\begin{array}{c} x^{\pm }+u^{\pm }\overset{r_{1}^{\pm }}{\to }x^{\pm }+u^{\pm }+y^{\mp }, \end{array}$$

where *y* ^∓^ replaces the original *y* ^±^.

#### Gain and Summation Blocks

A weighted summation block takes a number of input signals *u*_*i*_(*t*), *i* = 1, 2, …, *n* and produces an output signal $y(t)=\sum_{i=1}^{n}k_{i}u_{i}(t)$ for *k*_*i*_ ∈ ℝ. A gain block is a special weighted summation block with only one input *u*(*t*) that produces output *y*(*t*) = *k*_1_ *u*(*t*) for *k*_1_ ∈ ℝ. The gain block with *k*_1_ ≥ 0 can be realized by one pair of catalytic reactions,

$$\begin{array}{c} x^{\pm }+u^{\pm }\overset{r_{1}^{\pm }}{\to }x^{\pm }+u^{\pm }+y^{\pm }, \end{array}$$

one pair of degradation reactions,

$$\begin{array}{c} z^{\pm }+y^{\pm }\overset{r_{0}^{\pm }}{\to }z^{\pm }, \end{array}$$

and an annihilation reaction

$$\begin{array}{c} y^{+}+y^{-}\overset{r_{\text{gs}}}{\to }\emptyset , \end{array}$$

where *x* ^±^ and *z* ^±^ are auxiliary species. These reactions induce the following kinetic equations

$$\begin{array}{c} {\dot{y}}^{\pm }=r_{1}^{\pm }x^{\pm }u^{\pm }-r_{0}^{\pm }z^{\pm }y^{\pm }-r_{\text{gs}}\;y^{+}y^{-}. \end{array}$$

Let $k_{1}\equiv r_{1}^{+}x^{+}=r_{1}^{-}x^{-}$ and $k_{0}\equiv r_{0}^{+}z^{+}=r_{0}^{-}z^{-}$. The mass action of *y* becomes

$$\begin{array}{c} \dot{y}=k_{1}\left(u^{+}-u^{-}\right)-k_{0}\left(y^{+}-y^{-}\right)=k_{1}u-k_{0}y. \end{array}$$ (1)

When the steady state is reached, the changing rate of *y* (i.e., $\dot{y}$) equals zero, which implies $y=\frac{k_{1}}{k_{0}}u$. Note that the concentrations of the auxiliary species *x* ^±^ and *z* ^±^ are controlled externally. Therefore, it becomes theoretically possible to meet any required *k*_1_ by tuning the concentrations of these auxiliary species. For *k*_1_ < 0, the plus and minus signs of the superscripts of *y* in the pair of catalytic reactions should be swapped.

For the weighted summation block, the reactions are the same as those for the gain block except that the pair of catalytic reactions becomes

$$\begin{array}{c} x_{i}^{\pm }+u_{i}^{\pm }\overset{r_{i}^{\pm }}{\to }x_{i}^{\pm }+u_{i}^{\pm }+y^{\pm },\;\text{for}\;i=1,\ldots ,n. \end{array}$$

By setting $k_{i}\equiv r_{i}^{+}x_{i}^{+}=r_{i}^{-}x_{i}^{-}$ we obtain the equation

$$\begin{array}{c} y=\sum_{i}\frac{k_{i}}{k_{0}}u_{i} \end{array}$$ (2)

in the steady state. Similarly, if a scaling factor *k*_*i*_ < 0, we swap the signs in the superscript of *y* in the reaction corresponding to input *u*_*i*_.

It is worth noting that in contrast to the integration block, the weighted summation block only approximates the intended weighted summation when the steady state is not yet reached; this is often the case in practice. This approximation can be clearly seen from its transfer function $\frac{Y}{U}=\sum_{i}\frac{k_{i}}{s+k_{0}}$, rather than the ideal $\frac{Y}{U}=\sum_{i}\frac{k_{i}}{k_{0}}$. Although this approximation may seem to inevitably cause a chemical reaction implementation to deviate from its specification, we will later show methods that can be used to circumvent this imperfection.

#### Case Study

**Fig 2:**
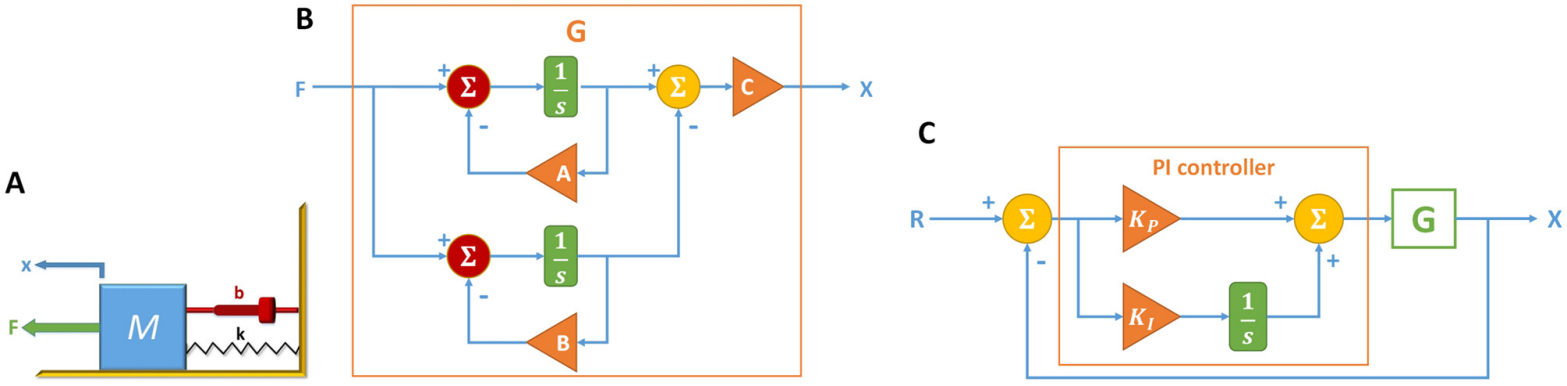
Mass-spring-damper system and its block diagrams. (A) MSD system. (Let *F* = 1 N, *M* = 1 kg, *b* = 10 N s/m, and *k* = 20 N/m.) (B) Block diagram of the MSD model, where the triangular blocks denote gain functions with their corresponding weights, the rectangular blocks denote integrators, and the circle blocks denote mixers for summation and/or subtraction. (Let *A* = 2.764, *B* = 7.236 and *C* = 0.2236.) (C) Block diagram of the PI-controlled MSD model, where *G* is the plant shown in (B). (Assume the values of *K*_*P*_ and *K*_*I*_ given in Table 2.)

To assess the benefit of the auxiliary species, we perform a proof-of-concept case study on the mass-spring-damper (MSD) system as shown in Fig 2A. The system can be modeled by the equation

$$M\ddot{x}+b\dot{x}+kx=F.$$

Using *M* = 1 kg, *b* = 10 N s/m, *k* = 20 N/m, and *F* = 1 N, by Laplace transform we derive the transfer function

$$G=\frac{1}{s^{2}+10s+20}\approx 0.2236\left(\frac{1}{s+2.764}-\frac{1}{s+7.236}\right)$$

The transfer function can be implemented with the block diagram shown in Fig 2B. The proportional-integral (PI) controller to the MSD system is shown in Fig 2C.

The block diagrams are constructed with *k*_0_ = 10 for all the summation and gain blocks, with the exception of the summation blocks shown in red and gain blocks *A* and *B* with *k*_0_ = 50. The values of *k*_*i*_’s are set to *αk*_0_, where the values of *α* are equal to the weights specified in the corresponding gain blocks. Additionally, we assume $q_{0}^{+}$ and $q_{0}^{-}$ have the same values as *k*_0_, and $q_{1}^{+}$ and $q_{1}^{-}$ are mismatched to *k*_1_ by 10%, where *q*_1_ and *q*_0_ are the rate constants of the catalytic and degradation reactions, respectively, formulated in the prior work [10].

**Fig 3:**
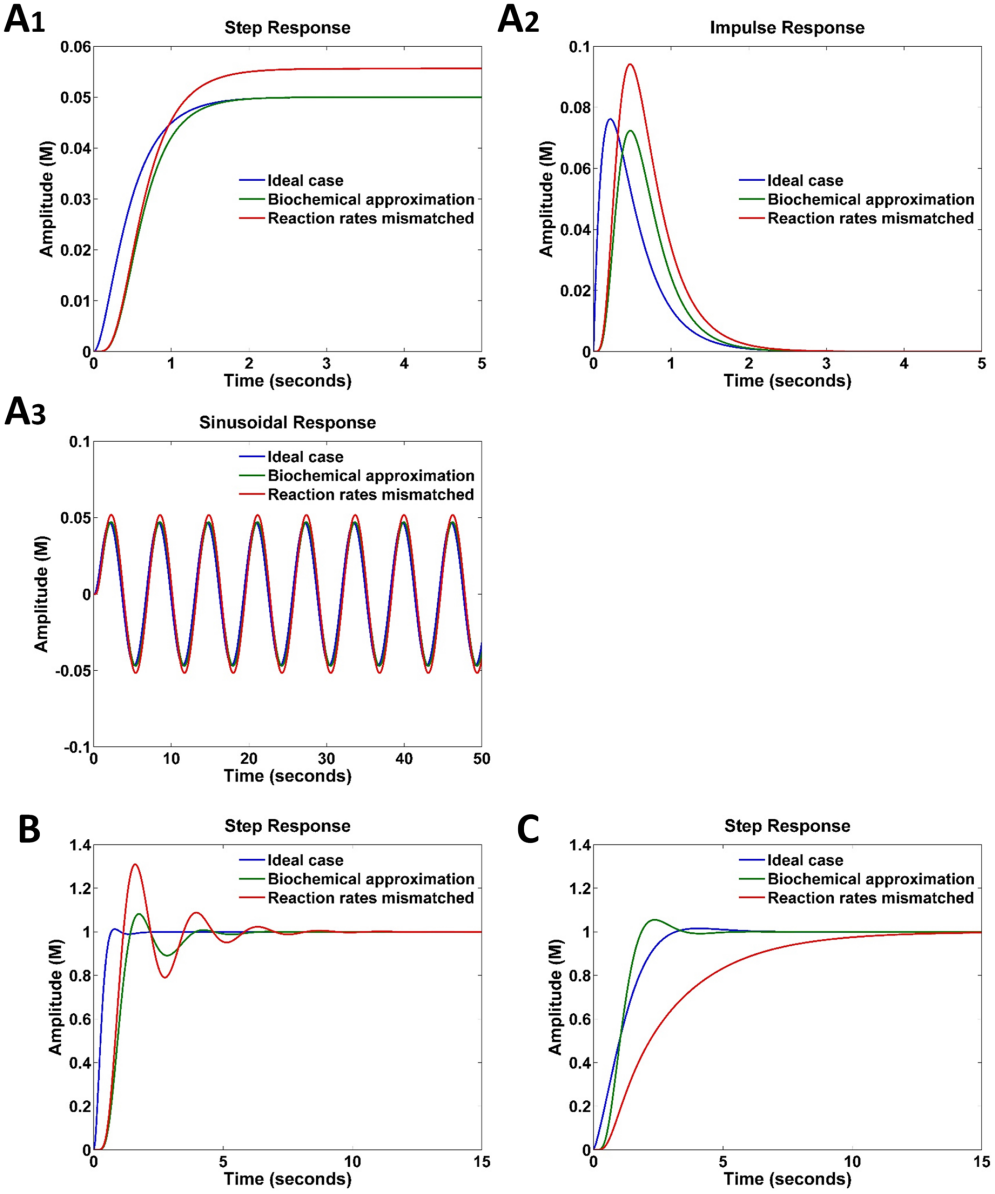
Responses of MSD systems with and without a PI controller. The blue, green, and red curves represent the responses in ideal, configurable biochemical implementation, and nonconfigurable biochemical implementation cases, respectively. (A) Step, impulse, and sinusoidal responses of the MSD. (B) Step responses of PI-controlled MSD. (Assume 10% rate mismatch in the MSD system.) (C) Step responses of PI-controlled MSD, where the MSD undergoes a parameter change with *b* = 40 N s/m and *k* = 60 N/m, respectively, to induce a gain change of *A* = 1.561, *B* = 38.44 and *C* = 0.0271.

Fig 3A1, 3A2, 3A3, and 3B show the step, impulse, and sinusoidal responses of the MSD system, and the step responses of the PI-controlled MSD system, respectively. Our method achieves better approximation to the ideal cases than the prior method [10]. One of the advantages of our method is that we can match the weight $\frac{k_{1}}{k_{0}}$ by tuning the concentrations of *x* ^±^ and *z* ^±^. In contrast, no tuning is possible in the prior method [10] to avoid the inexact gain $\frac{q_{1}}{q_{0}}$, where *q*_1_ and *q*_0_ are the rate constants of the catalytic and degradation reactions formulated in the prior work [10], respectively, due to the mismatch of the rate constants. (Note that the biochemical implementations have their own optimal *K*_*P*_ and *K*_*I*_ values, shown in Table 2, to approximate the ideal system.)

**Table 2:** Values of (*K*_*P*_, *K*_*I*_) in ideal, configurable and non-configurable implementations of the original and new MSD systems.

	Ideal	Config. Imp.	Non-config. Imp.
Original MSD	(30, 70)	(15, 20)	(15, 20)
New MSD	(15, 55)	(40, 60)	(15, 20)

Suppose that the spring and damper of the above MSD system are now replaced with new ones for *b* = 40 N s/m and *k* = 60 N/m. Our method can still adapt the PI-controller to the new MSD system without redesigning the PI-controller, whereas the prior method has no such capability. Because we can tune the concentrations of *x* ^±^ and *z* ^±^ in biochemical implementation, it is possible for us to adapt (*K*_*P*_, *K*_*I*_) to optimal values (40, 60) for the new PI-controlled MSD system, which is in contrast to the original system (15, 20). Fig 3C compares the results with and without such reconfigurability.

### DSD Realization of Configurable Primitive Components

We exploit the DNA strand displacement (DSD) technique [24, 33] as an experimental chassis for our synthesis flow, and map the synthesized CRNs to DSD reactions. The simulation is conducted using the Visual DSD tool [34] for validation.

To implement each primitive component, we map the reactions listed in Table 1 to DSD reactions considering the compatibility among the components. We adopt the two-domain DSD method [25] to compile CRNs into nucleic acid-based chemistry to achieve flexible composability. In this method, any free (i.e., unbound) single-stranded DNA species consist of two domains: one toehold domain and one recognition domain. A toehold may initiate the binding between a single-stranded DNA species and a double-stranded DNA species. The two-domain DSD method supports compositionality, which indicates that a block implemented with DSD reactions can be cascaded with other blocks implemented with DSD reactions. Thus, the output species of one block fits the input species required by its downstream blocks.

As a notational convention, we indicate a domain *t* to be a toehold by a hat “^” as $\hat{t}$. We also use superscript * to indicate the Watson-Crick complement of a domain DNA sequence (e.g., domain t* is sequence *CTAG* if domain *t* is sequence *GATC*). For a single-stranded species, we indicate the 5′ end using a vertical bar “∣” and the 3′ end by an angle bracket “〉” or “〈.” We call a single-stranded species of the form | 〉 (5′ end on the left and 3′ end on the right) and 〈 | (5′ end on the right and 3′ end on the left) an upper strand and a lower strand, respectively. A double-stranded species with an upper strand |*t* *u*〉 (and a lower strand 〈t* u*|) is denoted as [[*t* *u*]]. A double-stranded species with one or more dangling (or exposed) toeholds is called a gate, and is used to help transduce one or more species into other species. Double-stranded species without any dangling (exposed) toeholds or single-stranded species containing no toeholds are regarded as waste because they lack the ability to react with other species. The gates and species separately used to construct catalysis, degradation, and annihilation CRNs are called fuel.

**Fig 4:**
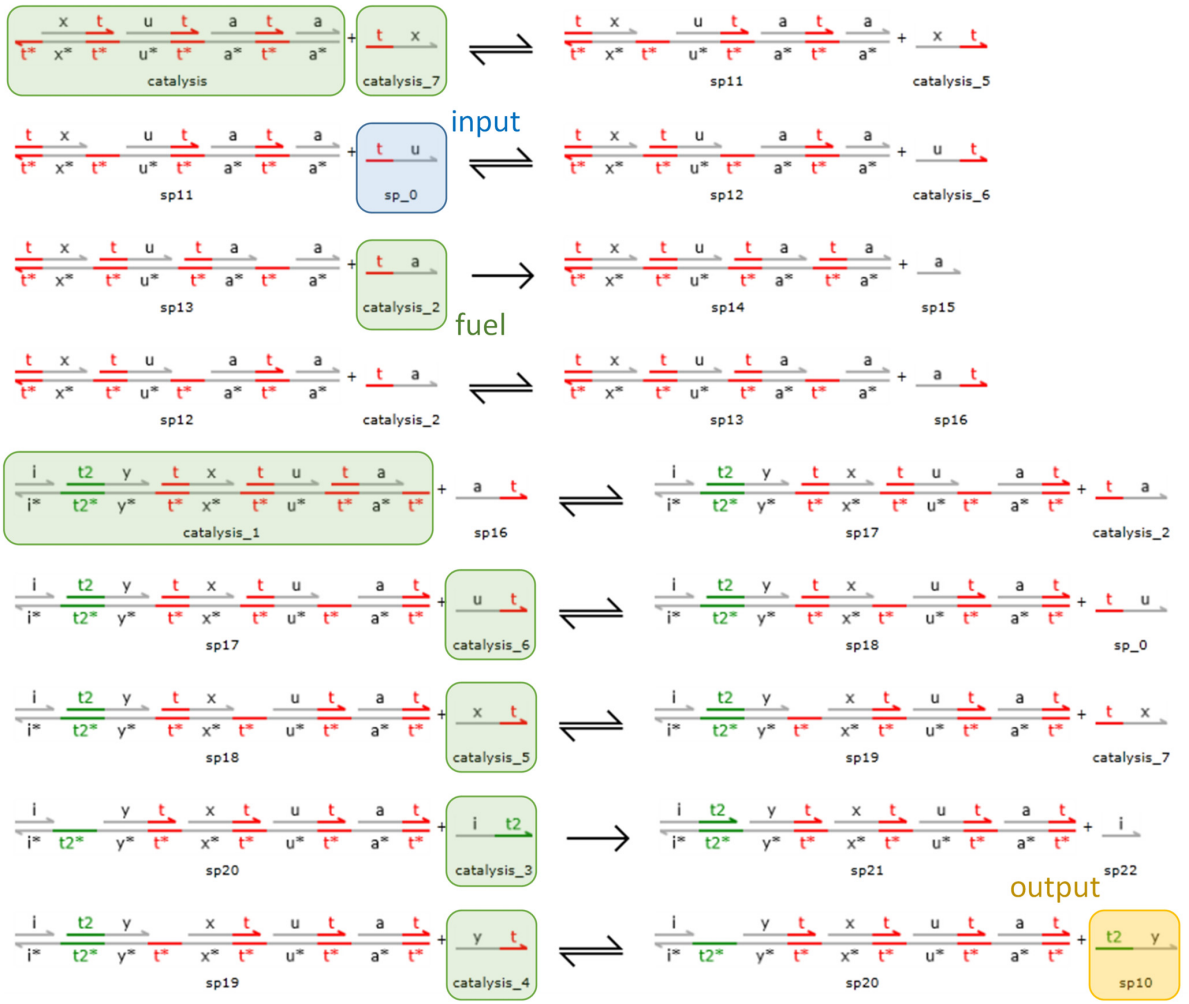
DSD realization of catalytic reactions. The species in green are fuels whose concentrations are fixed to 2000 nM to avoid the effect of fuel depletion. The species sp_0, sp10, and catalysis_7 represent the input *u* ^+^ or *u* ^−^, output *y* ^+^ or *y* ^−^, and catalyst *x* ^+^ or *x* ^−^, respectively. Species catalysis_7 reacts with the gate catalysis to begin the first stage of the reactions followed by a series of reactions involving species sp_0 and catalysis_2. At the end of the first stage, species sp16 is produced and reacts with the gate catalysis_1 to begin the second stage of the reactions. In this stage, species catalysis_7 and sp_0 are yielded to compensate for their consumption in the first stage. Finally, the output species sp10 is generated.

To ignore the effects of fuel depletion, we maintain all of the separately used species at constant concentrations. Moreover, we set the binding rates of all toeholds to 0.05 nM^−1^ s^−1^, which is consistent with the prior work [35]. We assume infinite unbinding rates [32, 36] to ignore the unproductive reactions [32] that result from the situation where a single-stranded species binds to a gate but does not further displace the strand with which it competes for the same recognition domain. Under these settings, we map the catalytic chemical reactions

$$\begin{array}{c} x^{\pm }+u^{\pm }\overset{r_{1}^{\pm }}{\to }x^{\pm }+u^{\pm }+y^{\pm } \end{array}$$

to the DSD reactions shown in Fig 4, where the domains highlighted in red and green are toeholds. Reactions $x^{+}+u^{+}\overset{r_{1}^{+}}{\to }x^{+}+u^{+}+y^{+}$ and $x^{-}+u^{-}\overset{r_{1}^{-}}{\to }x^{-}+u^{-}+y^{-}$ can be realized by the same set of DSD reactions except for species renaming (on recognition domains). For brevity, we simply use the same set of species $|\hat{t}\;x\rangle$ (*catalysis_7*), $|\hat{t}\;u\rangle$ (*sp_0*), and $|\hat{t_{2}}\;y\rangle$ (*sp10*) to denote the species *x* ^+^, *u* ^+^, and *y* ^+^, respectively, to implement the former reaction and to denote *x* ^−^, *u* ^−^, and *y* ^−^, respectively, for the latter reaction.

The set of DSD reactions outlined in Fig 4 consists of two stages. Gate *catalysis* initiates the first stage of the reactions by reacting with $|\hat{t}\;x\rangle$. At the end of the first stage, the species $|a\;\hat{t}\rangle$ will be produced, and this species will react with gate *catalysis_1* to initiate the second stage. During the second stage reactions, the species $|\hat{t}\;x\rangle$ and $|\hat{t}\;u\rangle$ will be produced to compensate for their consumption in the first stage.

We note that if the recognition domains of *u* and *x* were exchanged within the seven gates and one waste in the four first stage reactions, the entire set of reactions would still represent an implementation of the intended catalytic chemical reactions. However, the new arrangement of $\left[[u\;\hat{t}]\right]$ followed by $\left[[x\;\hat{t}]\right]$ would accelerate input $|\hat{t}\;u\rangle$ (effectively, *u* ^+^ or *u* ^−^ in the catalytic chemical reactions) consumption because the input $|\hat{t}\;u\rangle$ would directly react with the new *catalysis* gate. This is in contrast to the original *catalysis* structure, where $\left[[x\;\hat{t}]\right]$ is followed by $\left[[u\;\hat{t}]\right]$. Therefore, this consumption acceleration of $|\hat{t}\;u\rangle$ would amplify the bias between the production and consumption rates of $|\hat{t}\;u\rangle$. Because the concentration of $|\hat{t}\;u\rangle$ could not remain constant, the input species $|\hat{t}\;u\rangle$ could not be treated as a catalyst as expected. As a result, the new set of DSD reactions will not be as effective as the example presented in Fig 4.

**Fig 5:**
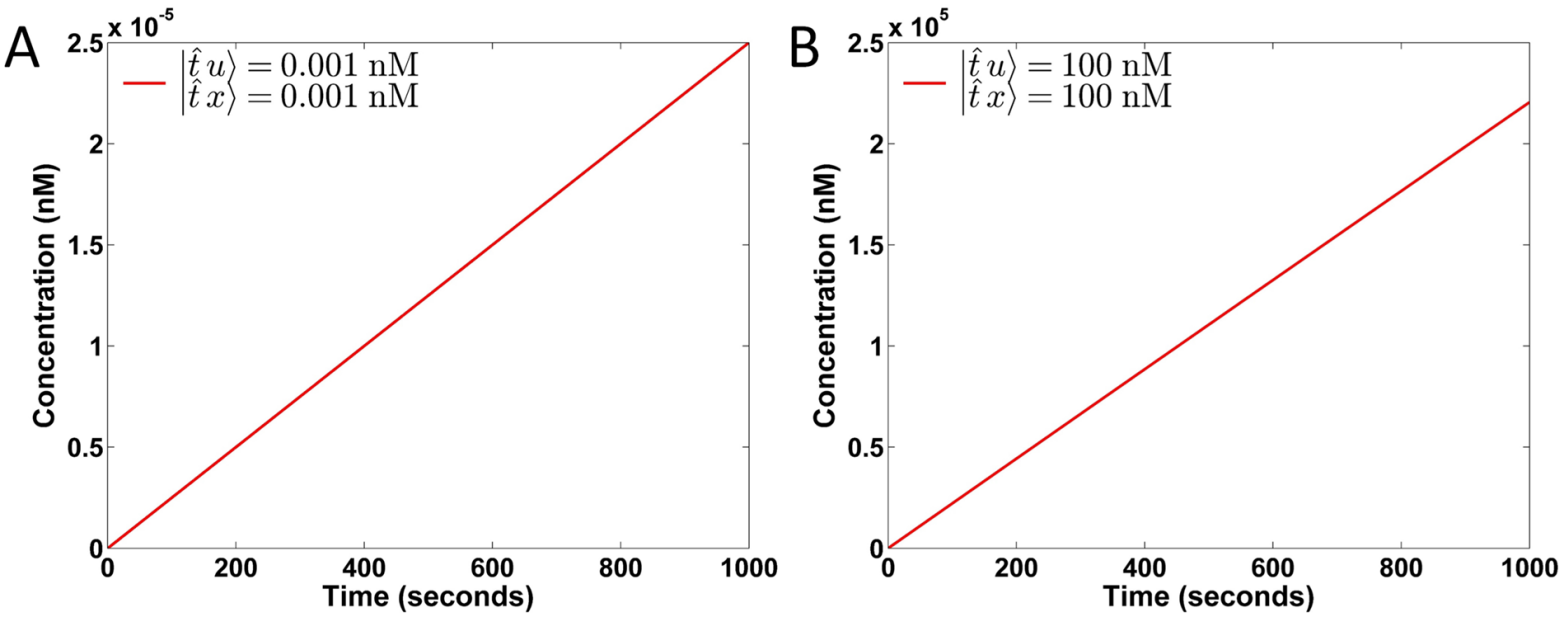
Concentration of $|\hat{t_{2}}\;y\rangle$ over time in DSD realized catalytic reactions. (A) $\left(|\hat{t}\;x\rangle ,|\hat{t}\;u\rangle )=(0.001,0.001\right)$ in nM and (B) $\left(|\hat{t}\;x\rangle ,|\hat{t}\;u\rangle )=(100,100\right)$ in nM.

To calibrate the values of the rate constants $r_{1}^{\pm }$, we conduct an experiment on the catalytic reaction $x^{\pm }+u^{\pm }\overset{r_{1}^{\pm }}{\to }x^{\pm }+u^{\pm }+y^{\pm }$. Let *x* ^±^ be mapped to $|\hat{t}\;x\rangle$, *u* ^±^ to $|\hat{t}\;u\rangle$ and *y* ^±^ to $|\hat{t_{2}}\;y\rangle$ at the concentrations summarized in Table 3. Additionally, we set and maintain the concentrations of all fuel to 2000 nM. However, the concentration of auxiliary species $|\hat{t}\;x\rangle$ can be obtained by the weight $k\equiv r_{1}^{+}x^{+}=r_{1}^{-}x^{-}$ after $r_{1}^{\pm }$ are determined. Based on the relationship $r_{1}^{\pm }x^{\pm }u^{\pm }=\dot{y^{\pm }}=y_{\text{final}}^{\pm }/\tau$, where $y_{\text{final}}^{\pm }$ are the concentrations of $|\hat{t_{2}}\;y\rangle$ after an elapsed time *τ* = 1000 seconds, we derive the rate constants $r_{1}^{\pm }$. As seen from Table 3, the reaction rates $r_{1}^{\pm }$ hold almost the same value when the concentrations of $|\hat{t}\;u\rangle$ and $|\hat{t}\;x\rangle$ are smaller than 100 nM, but slightly decrease when one of the concentrations approaches 100 nM. Because $|\hat{t}\;u\rangle$ and $|\hat{t}\;x\rangle$ will be low concentrations (approximately 10^−6^ to 1 nM) in our later experiments, we set $r_{1}^{\pm }=0.0249$ nM^−1^ s^−1^. Fig 5A and 5B show the concentration of $|\hat{t_{2}}\;y\rangle$ over time under the concentration (in nM) settings of $\left(|\hat{t}\;x\rangle ,|\hat{t}\;u\rangle \right)$ at (0.001, 0.001) and (100, 100), respectively. These results suggest that the DSD reactions produce the output *y* ^±^ (i.e., |*t*_2_ *y*〉) at a constant rate. The linearity of the DSD reactions indicates their adequacy in realizing our proposed configurable primitive components.

**Table 3:** Experimental data used for measuring the rate constant of DSD catalysis.

$\left(|\hat{t}\;u\rangle ,|\hat{t}\;x\rangle ,|\hat{t_{2}}\;y\rangle \right)$	$r_{1}^{\pm }$
(nM, nM, nM)	(nM^−1^ s^−1^)
(1, 1, 24.97)	0.0250
(1, 100, 2272)	0.0227
(10, 10, 2469)	0.0247
(100, 1, 2436)	0.0244
(100, 100, 220492)	0.02205
(10^−3^, 10^−3^, 2.49 × 10^−3^)	0.0249

**Fig 6:**
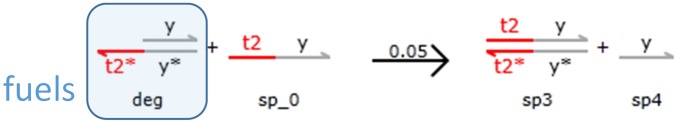
DSD realization of the degradation reaction. Here, deg serves as fuel with its concentration maintained at a fixed value and corresponds to catalysts *z* ^±^. In the reaction, the rate constant equals the toehold binding rate 0.05 nM^−1^ s^−1^.

**Fig 7:**
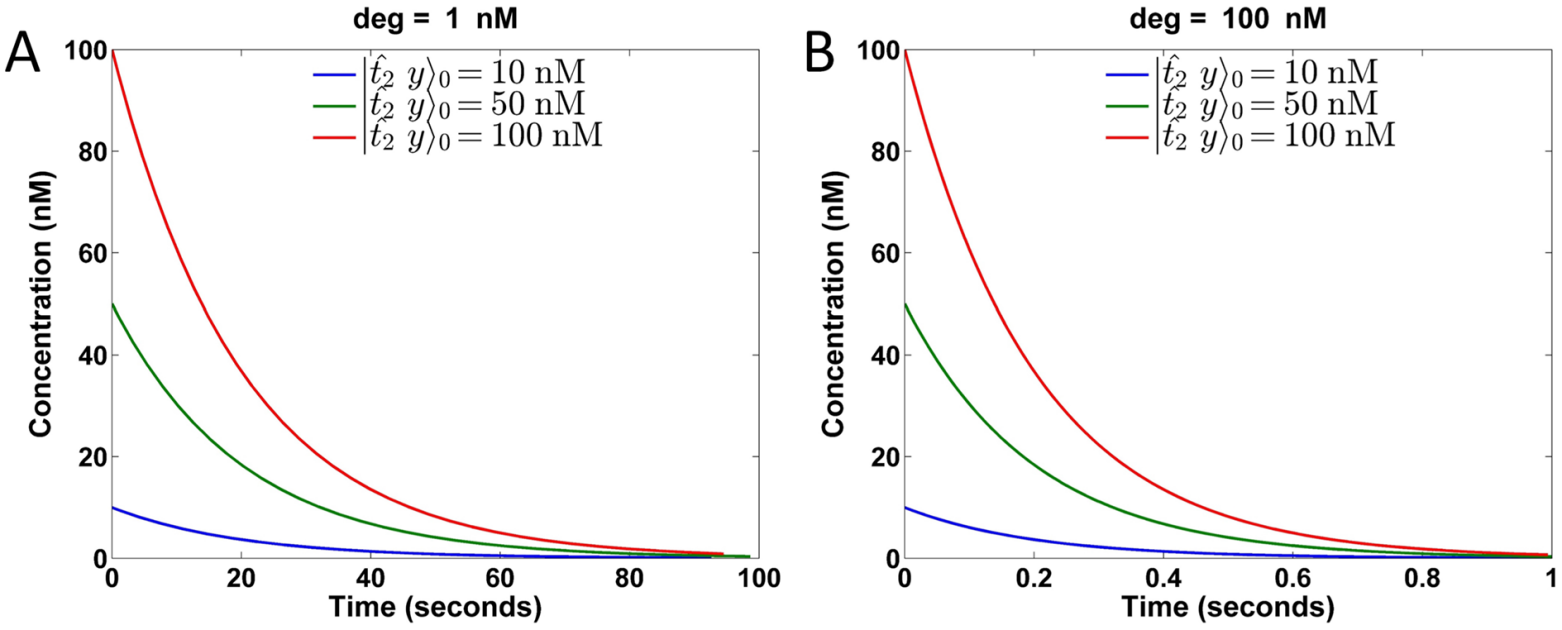
Simulation of DSD realized degradation reactions. (A) Concentration of $y^{\pm }=|\hat{t_{2}}\;y\rangle$ over time under deg = *z* ^±^ = 1 nM. (B) Concentration of $y^{\pm }=|\hat{t_{2}}\;y\rangle$ over time under deg = *z* ^±^ = 100 nM. The three curves in each case correspond to different initial concentrations *y*(0)^±^ = 10, 50, and 100 nM.

Similar to the construction proposed by Yordanov *et al.* when mapping chemical reactions $y^{\pm }\overset{q_{0}^{\pm }}{\to }\emptyset$ to DSD reactions [37], we map the degradation chemical reactions

$$\begin{array}{c} z^{\pm }+y^{\pm }\overset{r_{0}^{\pm }}{\to }z^{\pm }. \end{array}$$

to the DSD reactions shown in Fig 6. For brevity, the DSD reactions shown only correspond to one of the reactions $z^{+}+y^{+}\overset{r_{0}^{+}}{\to }z^{+}$ and $z^{-}+y^{-}\overset{r_{0}^{-}}{\to }z^{-}$. The species $\langle {\hat{t_{2}}}^{*}[[y]]$, labeled *deg*, is the only gate or fuel used in this network, while the species $|\hat{t_{2}}\;y\rangle$ represents *y* ^±^. The products *sp3* and *sp4* are waste products. In contrast to the DSD reactions proposed by Yordanov *et al.* [37] where the fuel concentrations are not maintained as constants, we maintain the fuel *deg* at a constant concentration. Therefore, the reaction can be effectively regarded as

$$deg+|\hat{t_{2}}\;y\rangle \;\overset{0.05}{\to }deg,$$

which is the same form as the above degradation chemical reactions. Thus, the species *deg* corresponds to *z* ^+^ or *z* ^−^, and the rate constants $r_{0}^{\pm }$ equal 0.05 nM^−1^ s^−1^, which is the binding rate constant of a toehold. By solving the differential equation of the kinetics of *y* ^±^, we derive that its concentration at time *t* is *y*(*t*)^±^ = *y*(0)^±^exp[−(0.05*z* ^±^)*t*], where *y*(0)^±^ are the initial concentrations of *y*(*t*)^±^. Fig 7A and 7B plot *y*(*t*)^±^ for *z* ^±^ = 1 nM and 100 nM, respectively. In each case, there are three curves corresponding to different initial concentrations of *y*(0)^±^ = 10, 50 and 100 nM. These six curves are shown in Fig 7A and 7B and exactly fit the ideal waveforms.

**Fig 8:**
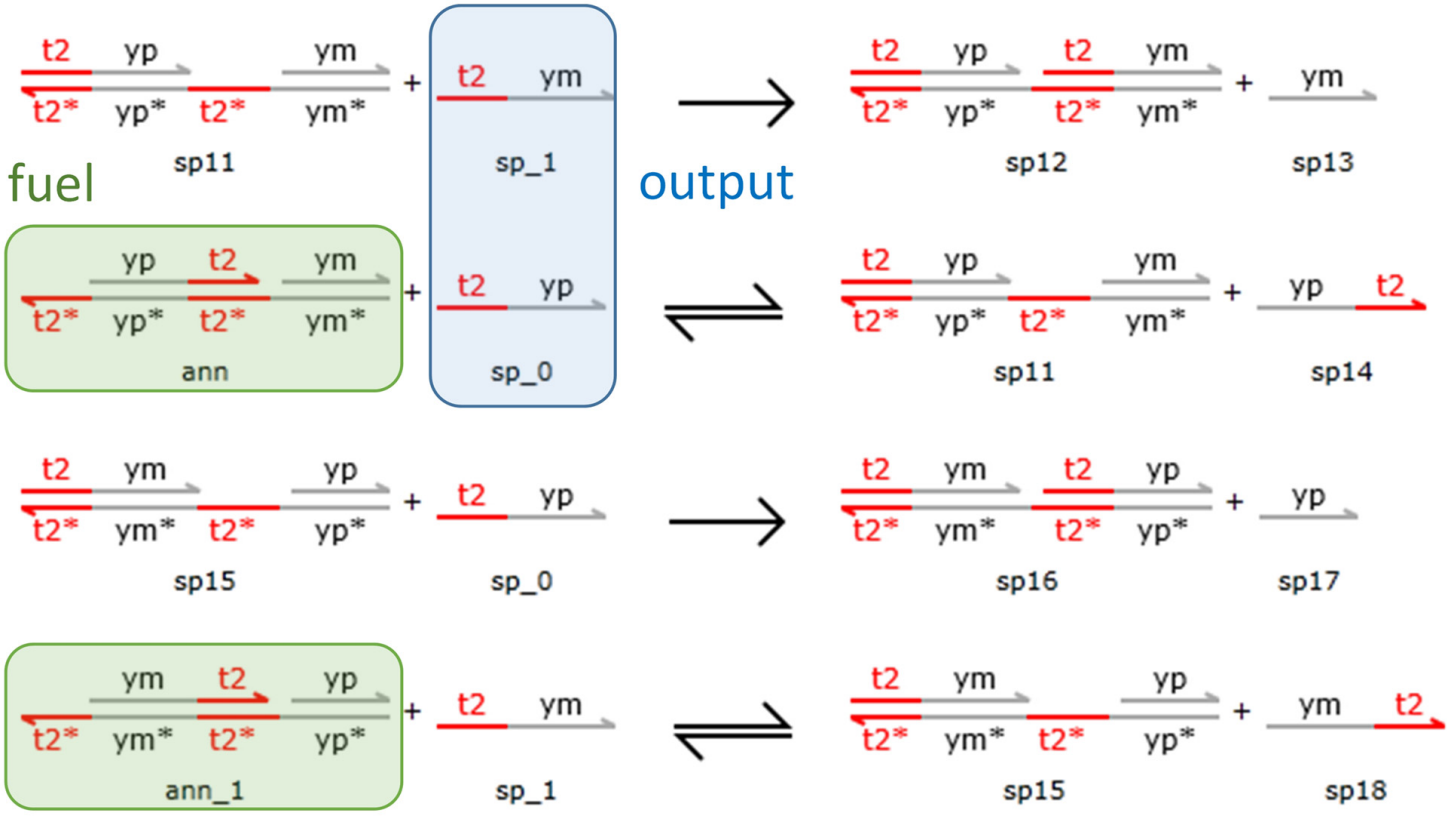
DSD realization of the annihilation reaction. Here, $|\hat{t_{2}}\;ym\rangle$ and $|\hat{t_{2}}\;yp\rangle$ correspond to the output species *y* ^+^ and *y* ^−^, respectively. Two fuels (ann and ann_1) with similar structures are used to ensure that the consumption rates of *y* ^+^ and *y* ^−^ are balanced.

Finally, following the construction scheme of Yordanov *et al.* [37], we map the annihilation chemical reaction

$$\begin{array}{c} y^{+}+y^{-}\overset{r_{\text{int}}\;\text{or}\;r_{\text{gs}}}{\to }\emptyset \end{array}$$ (3)

to the DSD reactions provided in Fig 8, where species $|\hat{t_{2}}\;yp\rangle$ and $|\hat{t_{2}}\;ym\rangle$ represent *y* ^+^ and *y* ^−^, respectively. These species correspond to the output species of the primitive components. Two fuels with similar structures are used to make the entire DSD reaction symmetric and balance the consumption rates of $|\hat{t_{2}}\;yp\rangle$ and $|\hat{t_{2}}\;ym\rangle$. As shown in Fig 8, the fuel *ann* first reacts with $|\hat{t_{2}}\;yp\rangle$ and then the product *sp11* reacts with $|\hat{t_{2}}\;ym\rangle$. In contrast, the fuel *ann_1* first reacts with $|\hat{t_{2}}\;ym\rangle$ followed by the reaction between the product *sp15* and $|\hat{t_{2}}\;yp\rangle$. Therefore, the consumption rates of $|\hat{t_{2}}\;yp\rangle$ and $|\hat{t_{2}}\;ym\rangle$ are balanced and achieve effective annihilation.

**Fig 9:**
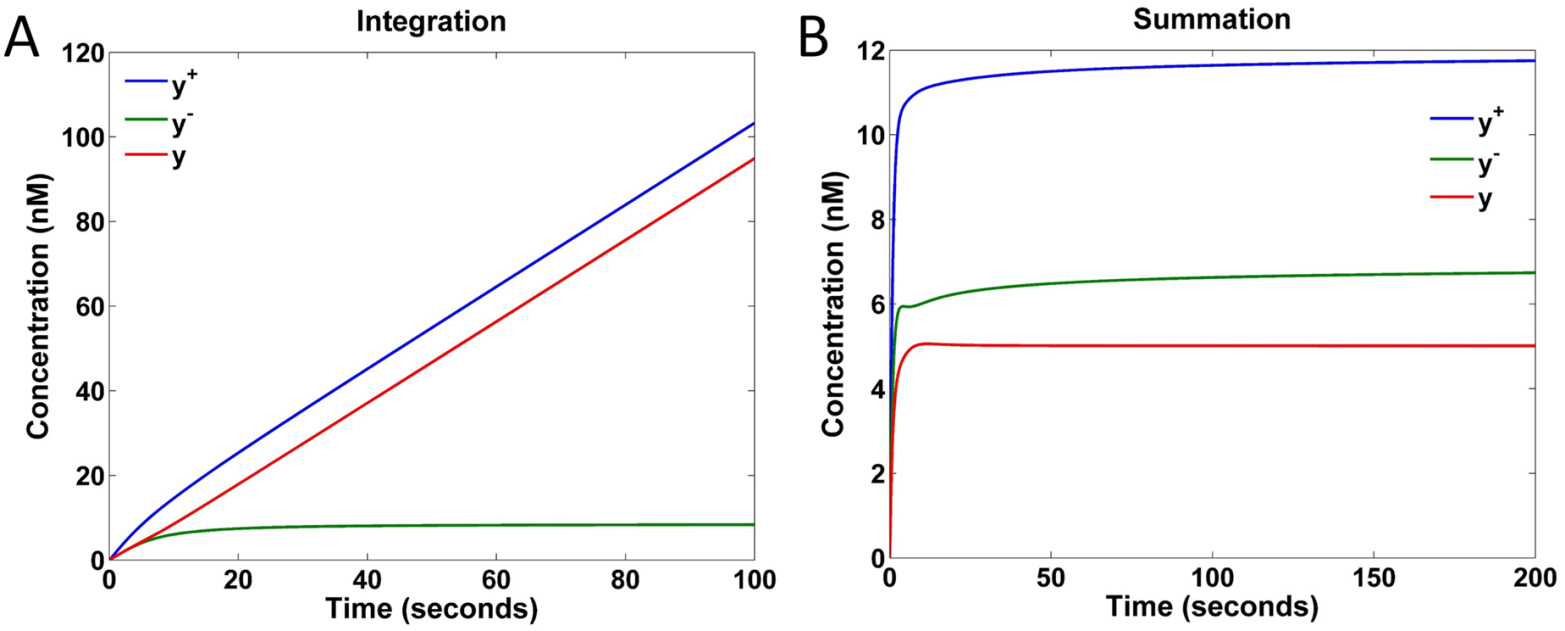
Simulation of DSD realized primitive components. (A) Concentrations of *y* ^+^, *y* ^−^, and *y* = *y* ^+^−*y* ^−^ of the integration component computing $y=\int_{0}^{t}u(\tau )\text{d}\tau$ under constant inputs *u* ^+^ = 2 and *u* ^−^ = 1 nM. (B) Concentrations of *y* ^+^, *y* ^−^, and *y* = *y* ^+^−*y* ^−^ of the weighted summation component computing *y* = 2*u*_1_ + *u*_2_ under constant inputs $\left(u_{1}^{+},u_{1}^{-})=(5,3\right)$ and $\left(u_{2}^{+},u_{2}^{-})=(2,1\right)$ in nM.

The above DSD reactions for catalysis, degradation, and annihilation form a set of basic elements that enable the construction of primitive components and any proper linear system. We built the proposed configurable primitive components of integration and weighted summation with DSD reactions. The simulation results are shown in Fig 9. Fig 9A shows the output response of the integration component computing $y=\int_{0}^{t}u(\tau )\text{d}\tau$ with respect to fixed inputs *u* ^+^ = 2 nM and *u* ^−^ = 1 nM. The concentration of *y* ^−^ (green curve) remains at approximately 8 nM due to the effect of the annihilation reaction [Eq (3)], while the concentration of *y* ^+^ (blue curve) grows to 105 nM after 100 seconds. As expected, the output signal *y* = *y* ^+^−*y* ^−^ grows linearly and reaches 97 nM after 100 seconds. Fig 9B shows the simulation results of the weighted summation component computing *y* = 2*u*_1_ + *u*_2_. Two sets of inputs $\left(u_{1}^{+},u_{1}^{-})=(5,3\right)$ and $\left(u_{2}^{+},u_{2}^{-})=(2,1\right)$ in nM are observed. As expected, the component functions correctly with *y* = 12 − 7 = 5 (for *y* ^+^ = 2 × 5 + 2 = 12 and *y* ^−^ = 2 × 3 + 1 = 7) nM.

The above simulation results confirm the successful realization of the integration and weighted summation components using DSD reactions. Because good compatibility is observed among the blocks constructed using the two-domain DSD method, these primitive DSD reactions can be composed to realize sophisticated linear systems. Later we will show that any proper transfer functions can be realized using the DSD realized primitive components.

### Implementation of Transfer Functions in Biochemistry

#### Transfer Function Decomposition

The transfer function *G*(*s*) of an LTI system can always be written as

$$\begin{array}{c} G=\frac{Y(s)}{U(s)}=\frac{b_{m}s^{m}+b_{m-1}s^{m-1}+\cdots +b_{0}}{a_{n}s^{n}+a_{n-1}s^{n-1}+\cdots +a_{0}}, \end{array}$$ (4)

where *Y*(*s*) and *U*(*s*) are the Laplace transforms of the output *y*(*t*) and input *u*(*t*) signals, respectively. A transfer function is called strictly proper, as we shall assume, if *m* < *n* (i.e., the degree of the numerator polynomial is smaller than the denominator polynomial).

Using the fundamental theorem of algebra, *U*(*s*) can be rewritten as

$$U\left(s\right)={\left(s+\alpha_{1}\right)}^{n_{1}}\cdots {\left(s+\alpha_{l}\right)}^{n_{l}}{\left(s^{2}+\beta_{1}s+\gamma_{1}\right)}^{m_{1}}\cdots {\left(s^{2}+\beta_{p}s+\gamma_{p}\right)}^{m_{p}},$$

where *α*_*i*_, *β*_*i*_ and *γ*_*i*_ are real numbers satisfying $\beta_{i}^{2}-4\gamma_{i}<0$ for stable LTI systems, *l* and *p* are non-negative integers, and *n*_*i*_ and *m*_*i*_ are positive integers. By partial fraction expansion, Eq (4) can be factorized as

$$G\left(s\right)=\sum_{i=1}^{l}\sum_{j=1}^{n_{i}}\frac{A_{ij}}{{\left(s+\alpha_{i}\right)}^{j}}+\sum_{i=1}^{p}\sum_{j=1}^{m_{i}}\frac{B_{ij}s+C_{ij}}{{\left(s^{2}+\beta_{i}s+\gamma_{i}\right)}^{j}},$$

where *A*_*ij*_, *B*_*ij*_, and *C*_*ij*_ are real coefficients. Because the terms of $\frac{1}{{\left(s+\alpha_{i}\right)}^{j}}$ and $\frac{1}{{\left(s^{2}+\beta_{i}+\gamma_{i}\right)}^{j}}$ can be realized by cascading *j* times the elementary modules of $\frac{1}{\left(s+\alpha_{i}\right)}$ and $\frac{1}{\left(s^{2}+\beta_{i}s+\gamma_{i}\right)}$, respectively, we only need to implement the set

$$\begin{array}{c} \{\frac{D_{1}}{s+\alpha },\frac{D_{2}}{s^{2}+\beta s+\gamma },\frac{D_{3}s}{s^{2}+\beta s+\gamma }\}, \end{array}$$ (5)

of elementary modules, where *D*_1_, *D*_2_ and *D*_3_ are real numbers. We refer to the three elementary modules as the “degree-(1, 0),” “degree-(2, 0),” and “degree-(2, 1)” modules, respectively, according to the degrees of their denominator and numerator polynomials.

Below we investigate how to implement these three elementary modules to construct any LTI system with CRNs. First, we provide a naive construction to illustrate inexactness, and then present a refined exact solution.

#### Naive Implementation of Elementary Modules

**Fig 10:**
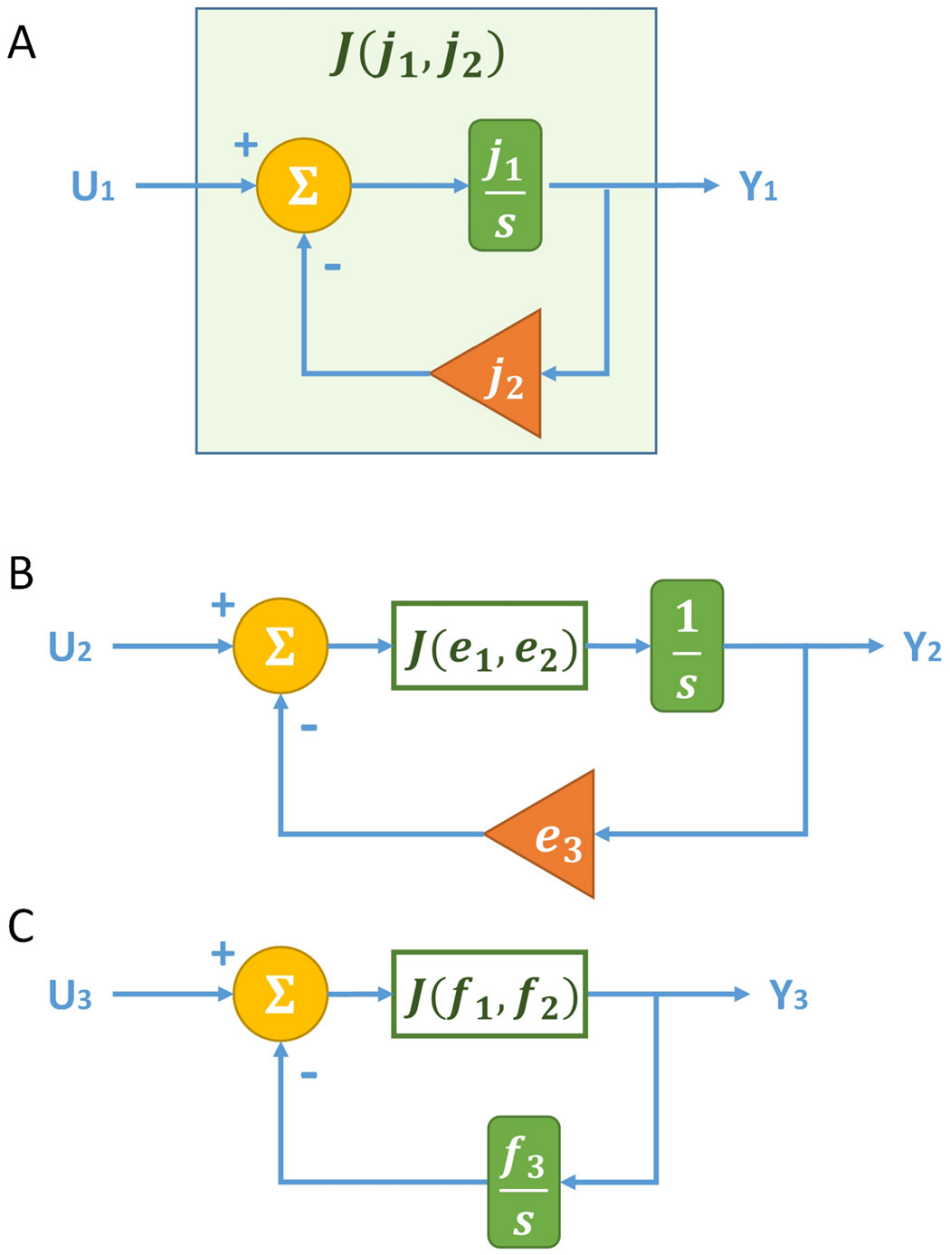
Block diagrams of naive implementation of elementary modules. (A) Block diagram of the degree-(1, 0) module with parametric weights *j*_1_ and *j*_2_ to match the transfer function coefficients. (B) Block diagram of the degree-(2, 0) module with parametric weights *e*_1_, *e*_2_, and *e*_3_ to match the transfer function coefficients. (C) Block diagram of the degree-(2, 1) module with parametric weights *f*_1_, *f*_2_, and *f*_3_ to match the transfer function coefficients.

To build a degree-(1, 0) module *J*(*j*_1_, *j*_2_) with parameters *j*_1_, *j*_2_ with real values, one might use the negative feedback loop consisting of a forward integration block of $\frac{j_{1}}{s}$ and a negative feedback of gain *j*_2_ (as shown in Fig 10A). The transfer function of this realization equals $\frac{j_{1}}{s+j_{1}j_{2}}$, which exactly represents the target form. Recall that the introduction of auxiliary species enables the configurability of primitive components, and thus the values of *j*_1_ and *j*_2_ can be set as desired.

To build a degree-(2, 0) module, one has to increase the degree of the denominator polynomial. Therefore, one might try to use a degree-(1, 0) module for a compositional construction (as shown in Fig 10B). Let the degree-(1, 0) module be *J*(*e*_1_, *e*_2_) fed to a forward integration block of $\frac{1}{s}$, and let the negative feedback have gain *e*_3_. Then, the transfer function of the block diagram equals $\frac{e_{1}}{s^{2}+e_{1}e_{2}s+e_{1}e_{3}}$ as desired.

To build a degree-(2, 1) module, one might also try to use a degree-(1, 0) module for compositional construction (as shown in Fig 10C). Let the degree-(1, 0) module be *J*(*f*_1_, *f*_2_) fed forward to the output, and place an integration block of $\frac{f_{3}}{s}$ on the negative feedback path. Then, the transfer function of the block diagram equals $\frac{f_{1}s}{s^{2}+f_{1}f_{2}s+f_{1}f_{3}}$ as desired.

Although the block diagrams of Fig 10 yield the desired transfer functions of the elementary modules, they are problematic due to the imperfection of the weighted summation components realized by the chemical reactions (with transfer function $\sum_{i}\frac{k_{i}}{s+k_{0}}$ instead of $\sum_{i}\frac{k_{i}}{k_{0}}$ as discussed earlier in the primitive component construction section). The following example demonstrates that a system constructed in this fashion may deviate drastically from the specification and can even be unstable.

Consider the LTI system specified by transfer function

$$\begin{array}{c} G=\frac{3s^{4}+19s^{3}+47s^{2}+51s+22}{s^{5}+10s^{4}+36s^{3}+67s^{2}+66s+30}. \end{array}$$ (6)

By partial fraction expansion, *G* can be expressed as

$$\begin{array}{c} G=\frac{1}{s^{2}+3s+3}+\frac{s}{s^{2}+2s+2}+\frac{2}{s+5}. \end{array}$$ (7)

By defining

$$\begin{array}{c} \left\{ \begin{array}{c} G_{1}\equiv \frac{1}{s^{2}+3s+3},\\ G_{2}\equiv \frac{s}{s^{2}+2s+2},\\ G_{3}\equiv \frac{2}{s+5}, \end{array}\right. \end{array}$$ (8)

they correspond to the basic elements in Eq (5) and can be implemented with the block diagrams of Fig 10 by setting (*j*_1_, *j*_2_) = (2, 2.5), (*e*_1_, *e*_2_, *e*_3_) = (1, 3, 3) and (*f*_1_, *f*_2_, *f*_3_) = (1, 2, 2). Assume that *k*_0_ = *a* for some positive real constant *a* for all weighted summation primitive components; then, assume that *k*_*i*_, *i* = 1, …, *n*, of a weighted summation primitive component are determined according to the required weights. The transfer functions after the chemical reaction realization become

$$\begin{array}{c} \left\{ \begin{array}{cc} G_{1}\to \frac{a^{2}\left(s+a\right)}{s^{2}{\left(s+a\right)}^{3}+3a^{2}s\left(s+a\right)+3a^{3}} & \equiv G_{1}^{\prime },\\ G_{2}\to \frac{a^{2}s}{s^{2}{\left(s+a\right)}^{2}+2a^{2}s+2a^{2}} & \equiv G_{2}^{\prime },\\ G_{3}\to \frac{2a(s+a)}{s{\left(s+a\right)}^{2}+5a^{2}} & \equiv G_{3}^{\prime }. \end{array}\right. \end{array}$$ (9)

Based on (asymptotic) stability analysis, the implemented system is unstable when a pole of the transfer functions (i.e., a root of the denominator polynomials) falls in the right-half of the *s*-plane. To avoid instability, the value of *a* has to be carefully determined. However, choosing a proper value for *a* can be tedious and sometimes even impossible.

**Fig 11:**
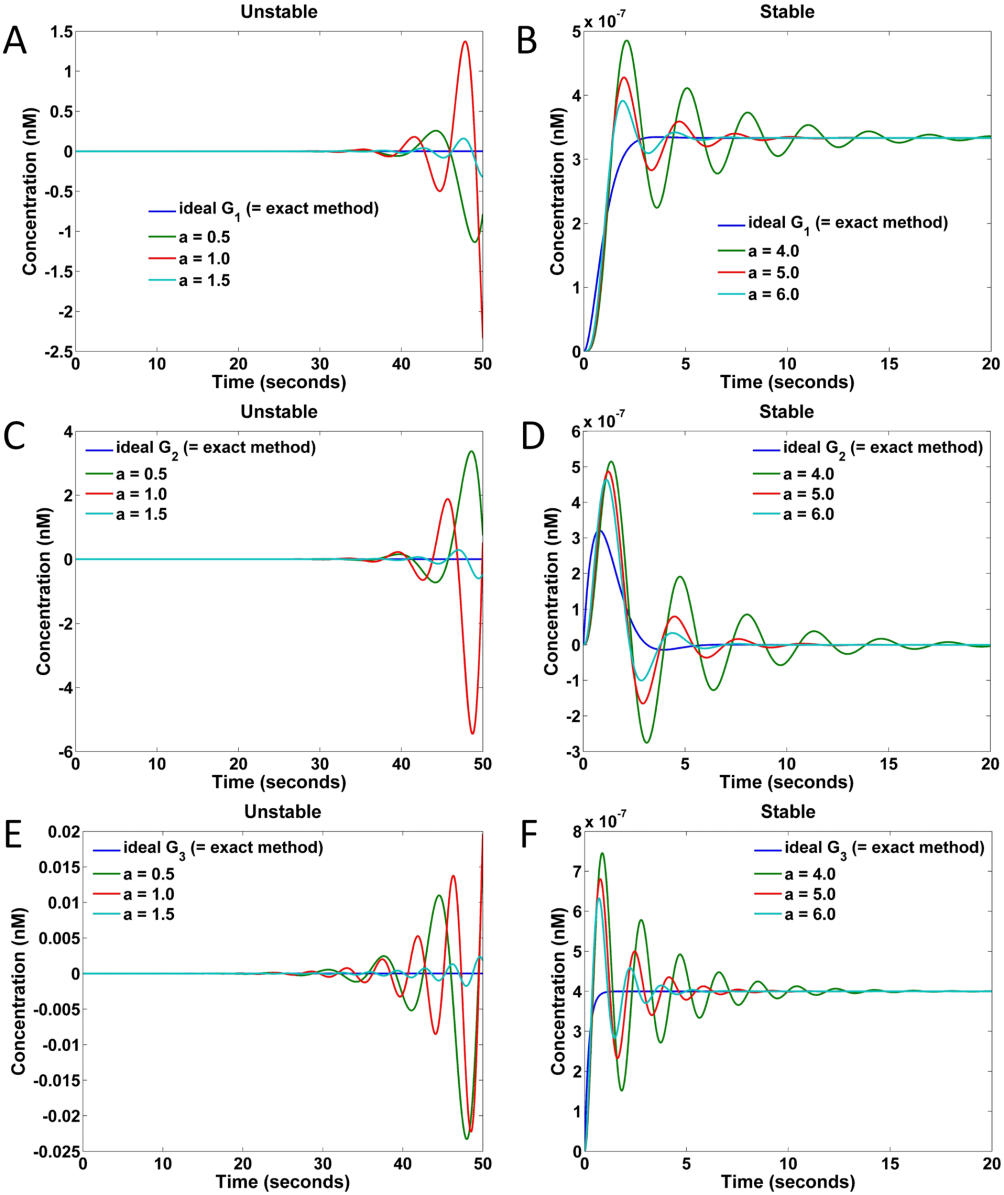
Responses of naive implementations to a step input of amplitude 10^−6^ nM under different values of parameter *a*. (A) Responses of $G_{1}^{\prime }$ under *a* = 0.5, 1.0, 1.5. (B) Responses of $G_{1}^{\prime }$ under *a* = 4.0, 5.0, 6.0. (C) Responses of $G_{2}^{\prime }$ under *a* = 0.5, 1.0, 1.5. (D) Responses of $G_{2}^{\prime }$ under *a* = 4.0, 5.0, 6.0. (E) Responses of $G_{3}^{\prime }$ under *a* = 0.5, 1.0, 1.5. (F) Responses of $G_{3}^{\prime }$ under *a* = 4.0, 5.0, 6.0.

The responses of $G_{1}^{\prime }$, $G_{2}^{\prime }$, and $G_{3}^{\prime }$ to the step input with an amplitude of 10^−6^ are shown in Fig 11. Specifically, Fig 11A and 11B show the responses of $G_{1}^{\prime }$, C and D show the responses of $G_{2}^{\prime }$, and E and F show the responses of $G_{1}^{\prime }$. As seen from the plots of Fig 11A, 11C, and 11E, the responses are unstable under the chosen small *a* values, while for plots of B, D, and F, the responses are stable under the chosen large *a* values. However, even the stable responses may deviate from the ideal transfer function responses to some extent. When *a* in $G_{1}^{\prime }$, $G_{2}^{\prime }$ and $G_{3}^{\prime }$ increases to 6, the maximal concentrations of the auxiliary species used in each case are 6 × 3/0.0249 ≈ 720, 6 × 2/0.0249 ≈ 480, and 6 × 2.5/0.0249 ≈ 600 nM, respectively, where the factors 3, 2 and 2.5 are the weights of the gain blocks in each system. As seen in Fig 11, the naive implementations cannot approximate the ideal responses well even under such high concentrations.

#### Exact Implementation of Elementary Modules

To prevent the non-ideal effect of the weighted summation components, we present a refined method that exactly implements the three elementary modules with chemical reactions.

**Fig 12:**
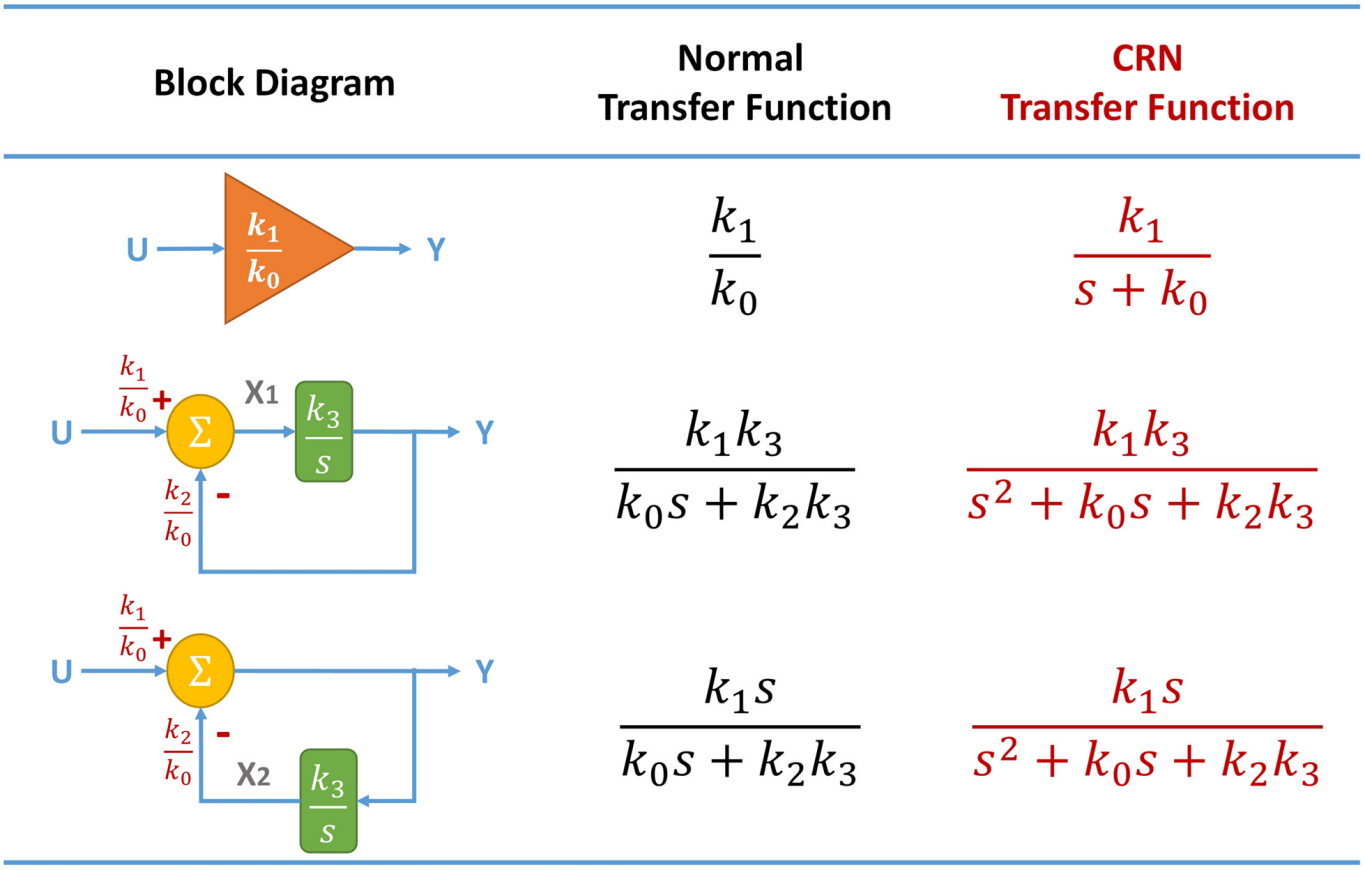
Block diagrams and their transfer functions for the exact implementation of the elementary modules. The first, second, and third rows in the table correspond to the implementations of the degree-(1, 0), degree-(2, 0), and degree-(2, 1) modules, respectively. The first, second, and third columns in the table show the block diagrams, normal transfer functions, and CRN transfer functions, respectively. A normal transfer function is obtained directly from its block diagram, whereas a CRN transfer function is obtained when the gain and summation blocks in the block diagram are realized using the gain and summation CRNs in Table 1.

The degree-(1, 0) module can be realized directly by the CRN transfer function of the primitive gain block. For the other two modules, we take advantage of the non-ideal form of the weighted summation block and devise their corresponding block diagrams, whose CRN transfer functions match the specification. The block diagrams, normal transfer functions, and CRN transfer functions for the three elementary modules are summarized in Fig 12. The CRN transfer function of the degree-(2, 0) module can be calculated from

$$\begin{array}{c} \left\{ \begin{array}{c} X_{1}=\frac{k_{1}}{s+k_{0}}U-\frac{k_{2}}{s+k_{0}}Y,\\ Y=\frac{k_{3}}{s}X_{1}. \end{array}\right. \end{array}$$ (10)

Therefore, the CRN transfer function $\frac{Y}{U}$ equals

$$\frac{Y}{U}=\frac{k_{1}k_{3}}{s^{2}+k_{0}s+k_{2}k_{3}}.$$

Note that if the gain and summation blocks are ideal, the normal transfer function should be $\frac{k_{1}k_{3}}{k_{0}s+k_{2}k_{3}}$. Similarly, the CRN transfer function of the degree-(2, 1) module can be derived as

$$\frac{Y}{U}=\frac{k_{1}s}{s^{2}+k_{0}s+k_{2}k_{3}}.$$

As a result, the responses can be made exact through the control of auxiliary species concentrations.

There are two special cases that need attention. First, if the coefficient *β* vanishes in the denominators of the transfer functions $\frac{D_{2}}{s^{2}+\beta s+\gamma }$ and $\frac{D_{3}s}{s^{2}+\beta s+\gamma }$ of the degree-(2, 0) and degree-(2, 1) modules, respectively, we have to make *k*_0_ = 0 in the CRN transfer functions. Therefore, we need to remove the degradation reactions $z^{\pm }+y^{\pm }\overset{r_{0}^{\pm }}{\to }z^{\pm }$ from the summation blocks. Second, for the negative parameter *k*_1_ or *k*_2_ in Fig 12, as mentioned previously the plus and minus signs of the superscripts of *y* in the pair of catalytic reactions of the weighted summation CRN should be swapped; for negative *k*_3_, the superscript signs of *y* in the pair of catalytic reactions of the integration CRN should be swapped. Notice that *k*_0_ can always be non-negative for stable linear systems, whose poles should all be on the left-half of the *s*-plane. Therefore, with these modifications our method is sufficient to implement any stable linear system.

### DSD Realization of Transfer Functions

We map the naive and exact implementations of the transfer function specification of Eq (6) into DSD reactions for comparison. In the simulation, we assume the rate constants $r_{0}^{\pm }=0.05$ nM^−1^ s^−1^ and $r_{i}^{\pm }=0.0249$ nM^−1^ s^−1^ for *i* = 1, …, *n*.

**Fig 13:**
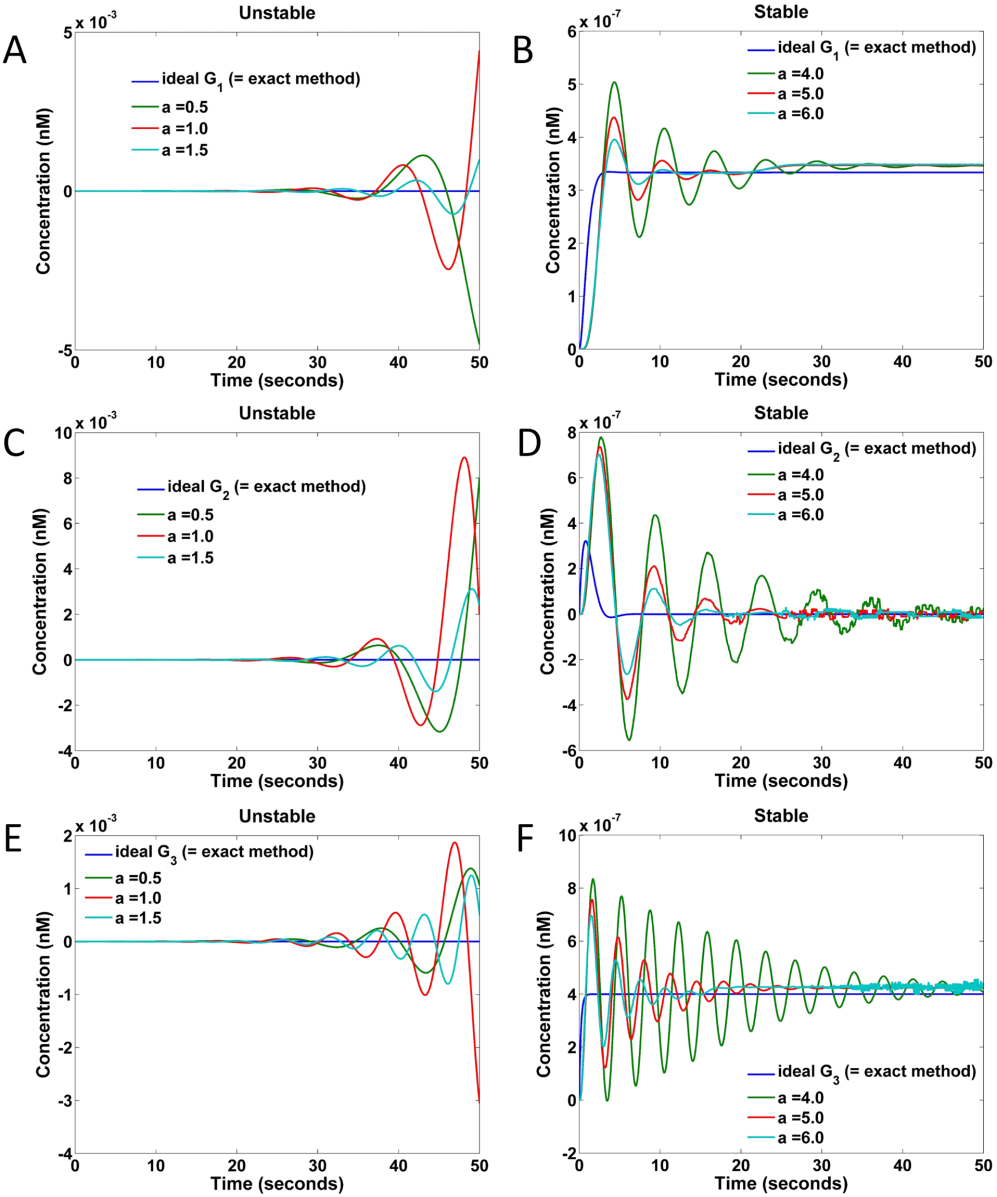
Responses of DSD realized naive implementations to a step input of amplitude 10^−6^ nM under different values of parameter *a*. (A) Responses of $G_{1}^{\prime }$ under *a* = 0.5, 1.0, 1.5. (B) Responses of $G_{1}^{\prime }$ under *a* = 4.0, 5.0, 6.0. (C) Responses of $G_{2}^{\prime }$ under *a* = 0.5, 1.0, 1.5. (D) Responses of $G_{2}^{\prime }$ under *a* = 4.0, 5.0, 6.0. (E) Responses of $G_{3}^{\prime }$ under *a* = 0.5, 1.0, 1.5. (F) Responses of $G_{3}^{\prime }$ under *a* = 4.0, 5.0, 6.0.

The responses of DSD that realized naive implementation of the transfer function specification are shown in Fig 13. Specifically, Fig 13A and 13B show the results realizing the transfer function *G*_1_ in Eq (8), C and D show those realizing *G*_2_, and E and F show those realizing *G*_3_. In the plots of Fig 13A, 13C, and 13E, the realized systems are unstable under the chosen small *a* values, while in the plots of B, D, and F the realized systems are stable under the chosen large *a* values. These results are consistent with the CRN responses of Eq (9) in Fig 11.

The maximum *k*_*i*_’s in the DSD realizations of $G_{1}^{\prime }$, $G_{2}^{\prime }$, and $G_{3}^{\prime }$ are 6.0 × 3 = 18.0, 6.0 × 2 = 12.0, and 6.0 × 2.5 = 15.0, respectively. Because the rate constants $r_{i}^{\pm }$, *i* = 1, …, *n* are set to 0.0249 nM^−1^ s^−1^, the corresponding concentrations of the catalysts *x* ^±^ in the gain and summation blocks in Fig 10 of $G_{1}^{\prime }$, $G_{2}^{\prime }$, and $G_{3}^{\prime }$ are approximately 18.0/0.0249 ≈ 720, 12.0/0.0249 ≈ 480, and 15/0.0249 ≈ 600 nM, respectively. Not only are such high concentrations undesirable, but it is also difficult to find a proper *a* value to fit the ideal response.

**Fig 14:**
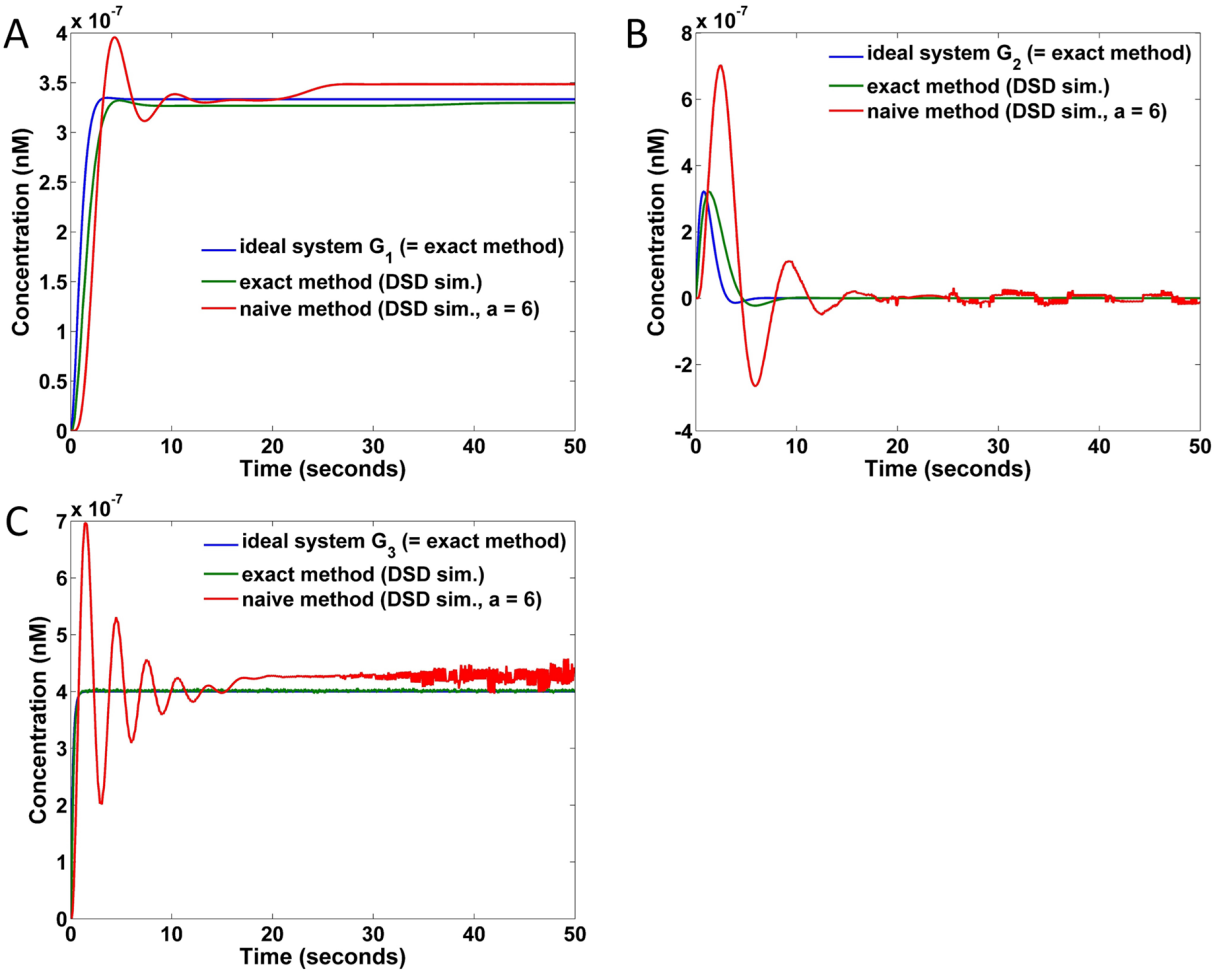
Response comparisons on DSD realized systems under the naive and exact implementations of transfer functions *G*_1_, *G*_2_, and *G*_3_. (A) Responses of *G*_1_ implementations. (B) Responses of *G*_2_ implementations. (C) Responses of *G*_3_ implementations. The curves in red corresponds to naive implementations (under the parameter setting *a* = 6 in $G_{i}^{\prime }$), while the curves in green correspond to exact implementations.

To compare the naive and exact implementations of transfer function specifications under DSD realization, the responses of the targeted transfer functions *G*_1_, *G*_2_ and *G*_3_ are plotted in Fig 14A, 14B, and 14C, respectively. The red curves in Fig 14A, 14B, and 14C correspond to the naive implementations with *a* = 6; the involved concentrations of catalysts are approximately 720, 480, and 600 nM, respectively. The species with the largest concentrations used in the exact implementations of *G*_1_ and *G*_2_ are both the catalysts *x* ^±^ in the integration CRNs. Because the weights of the integration blocks in the exact implementations of *G*_1_ and *G*_2_ are both equal to 2.0, the corresponding concentrations of *x* ^±^ in the catalytic reactions are approximately 80 nM. In contrast, in the exact implementation of *G*_3_ the catalysts *z* ^±^ in the gain block (where *k*_0_ equals 5.0) have the highest concentration (5.0/0.05 = 100 nM). Compared to the naive implementations involving concentrations as large as 720 nM, the exact implementations require much lower concentrations, and yet fit the ideal responses much better.

**Fig 15:**
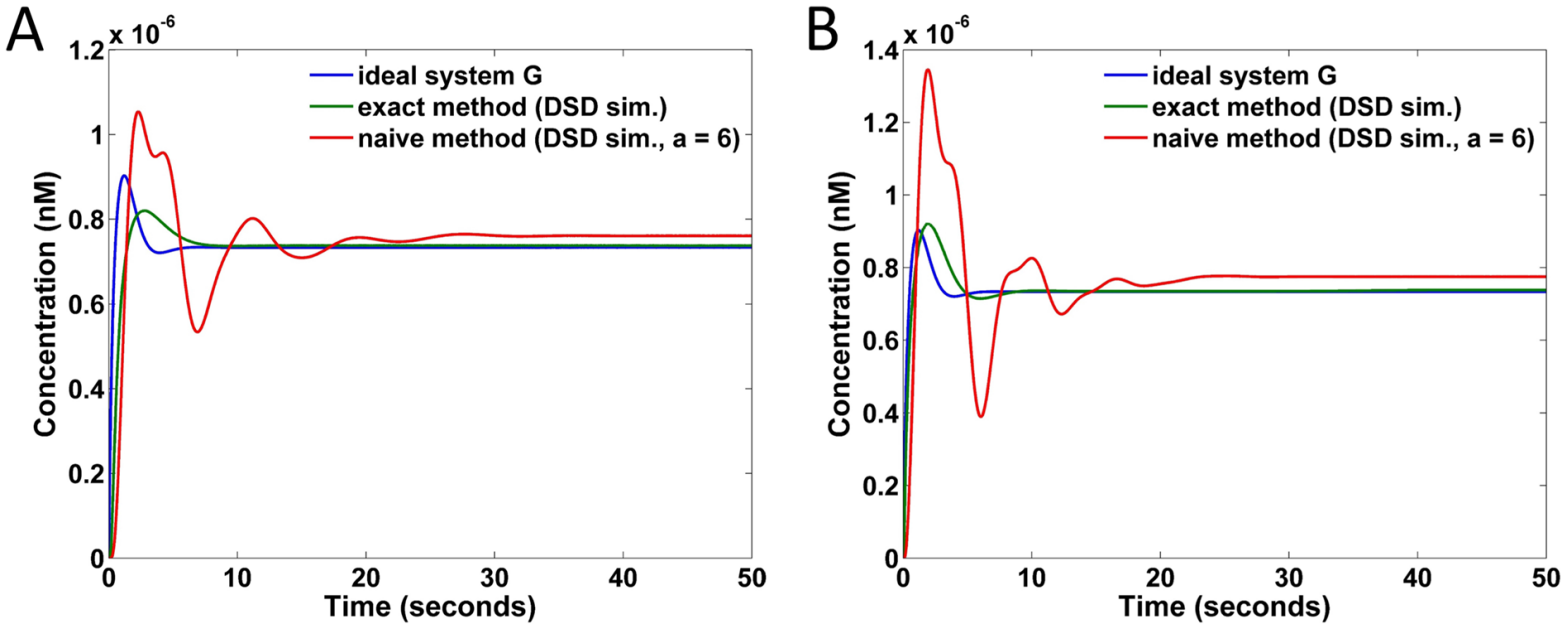
Response comparisons on DSD realized systems for the naive and exact implementations of transfer function *G*. (A) *G* implemented by a summation block adding *G*_1_, *G*_2_, and *G*_3_, assuming *a* = 6 for this summation block in both the naive and exact methods. (B) *G* implemented by superposing *G*_1_, *G*_2_, and *G*_3_, assuming that the DSD output species of *G*_1_, *G*_2_, and *G*_3_ have a common sub-domain that is recognized as the output of *G*.

For the overall transfer function *G* = *G*_1_ + *G*_2_ + *G*_3_, the responses of the DSD reactions realized via the naive and exact implementations are compared in Fig 15 against the ideal response. There are two approaches combining the subsystems *G*_1_, *G*_2_, and *G*_3_. One approach is to use a summation block to sum up the outputs *Y*_1_, *Y*_2_, and *Y*_3_ of *G*_1_, *G*_2_, and *G*_3_, respectively; the other approach is to keep *Y*_1_, *Y*_2_, and *Y*_3_ intact without summation by assuming that the output species of *Y*_1_, *Y*_2_, and *Y*_3_ share the same piece of the DNA segment (rather than the entire DNA segment) that corresponds to the intended final product. The result of the former approach is shown in Fig 15A and the latter approach is shown in Fig 15B, which corresponds to the direct superposition of individual *G*_1_, *G*_2_, and *G*_3_ responses. We observe that summing *Y*_1_, *Y*_2_, and *Y*_3_ with an additional summation block may result in slight distortions in comparison with direct superposition. In either case, the exact implementation fits the ideal response much better than the naive implementation.

## Discussion

### Rate Matching and Configurability

The CRN of the integration block proposed by Oishi and Klavins contains the catalytic reactions $u^{\pm }\overset{q_{c}^{\pm }}{\to }u^{\pm }+y^{\pm }$. The integration is made possible under the assumption that $q_{c}^{+}=q_{c}^{-}\equiv w_{\text{int}}$. Accordingly $\dot{y}=w_{\text{int}}\dot{u}$ (i.e., output *y* is a weighted integration of input *u*). However, the CRNs of the gain block contain one pair of additional degradation reactions $y^{\pm }\overset{q_{d}^{\pm }}{\to }\emptyset$. The gain function with weight *w*_gn_ is realized by assuming $\frac{q_{c}^{+}}{q_{d}^{+}}=\frac{q_{c}^{-}}{q_{d}^{-}}=w_{\text{gn}}$. Thus, not only $q_{c}^{+}=q_{c}^{-}$ but also $q_{d}^{+}=q_{d}^{-}=q_{c}^{+}/w_{\text{gn}}$ must be satisfied.

To cope with the problem of matching $q_{c}^{+}$ and $q_{c}^{-}$ in the integration CRN, Oishi and Klavins provided a solution by maintaining a large rate constant *q*_*ua*_ in the annihilation reaction $u^{+}+u^{-}\overset{q_{ua}}{\to }\emptyset$ so that $q_{c}^{+}$ and $q_{c}^{-}$ do not need to be exactly the same, as long as they are close enough to *w*_int_. The reason is that one of the concentrations of *u* ^+^ and *u* ^−^ would approach zero under fast annihilation, and thus $\dot{y}$ equals either *w*_int_ *u* ^+^ or *w*_int_ *u* ^−^. Because $q_{c}^{+}$ and $q_{c}^{-}$ are close to *w*_int_, the integration can be reasonably approximated. Similarly, the matching problem in the gain CRN is overcome by asserting not only a large *q*_*ua*_, but also a large *q*_*ya*_ in $y^{+}+y^{-}\overset{q_{ya}}{\to }\emptyset$. Thus, by requiring that $q_{c}^{\pm }$ are close enough and the ratios $\frac{q_{c}^{+}}{q_{d}^{+}}$ and $\frac{q_{c}^{-}}{q_{d}^{-}}$ are close to *w*_gn_, the gain function can be well approximated.

However, the above assumptions of fast annihilations $u^{+}+u^{-}\overset{q_{ua}}{\to }\emptyset$ (and $y^{+}+y^{-}\overset{q_{ya}}{\to }\emptyset$) may not be easily satisfied, especially when these reactions are achieved by composing multiple reactions (e.g., in the DSD realization). Two undesirable situations might occur in composite reactions even if the rate constant *q*_*ua*_ is high. One is that *u* ^+^ and *u* ^−^ both degrade, but the species with the lower concentration converges to a non-zero concentration value due to the restoration of the reactants by other reactions. The other is that although the species with the lower concentration vanishes, the remaining species converges to a concentration value less than ∣*u* ^+^−*u* ^−^∣ because some fuels can react with the remaining species. The approximations may be unsound when these situations occur. Although the effect of inappropriate assumptions might not be serious for a single primitive component, for a complex system composed of many primitive components the approximation would be crude due to error accumulations.

In contrast, because our primitive components are configurable due to the addition of the auxiliary species, we can match not only the reaction rates but also the required weights by tuning the concentrations of auxiliary species. Another advantage provided by the use of auxiliary species is the ability for dynamic system adaptation. As discussed in the case study of the PI-controller, the parameters of a PI-controller may be modified by simply tuning the concentrations of auxiliary species to react to environmental changes. Thus, by using auxiliary species we may fulfill a new system with the same set of reactions by tuning only the concentrations of the auxiliary species. In contrast, without auxiliary species the system would have to be redesigned by finding new catalysts and degradations to match the new weights and rate constants.

### Transfer Function Decomposition

A transfer function can be decomposed in many different ways. Two common choices are through parallel decomposition (*G* = *G*_1_ + *G*_2_) and serial decomposition (*G* = *G*_1_ ⋅ *G*_2_). In this paper, we adopt the former for the following reasons. First, given a strictly proper transfer function it is not always possible to decompose it as a product of the strictly proper transfer functions. For instance, the transfer function $\frac{2s^{2}+3s+4}{\left(s+1)(s+2)(s+3\right)}$ cannot be decomposed serially into a system involving the cascade of two strictly proper subsystems. Because only proper systems where the degree of the numerator polynomial does not exceed that of the denominator polynomial are physically realizable, the only hope for implementing the system physically is to have a product of bi-proper subsystems, where the degrees of the numerator and denominator polynomials are the same. For example, it can be decomposed as $\frac{2s^{2}+3s+4}{\left(s+1)(s+2\right)}\cdot \frac{1}{\left(s+3\right)}$. However, if we implement the system with CRNs or the primitives introduced previously, the bi-proper subsystem might be an approximation due to $\frac{2s^{2}+3s+4}{\left(s+1)(s+2\right)}=2-\frac{3s}{s^{2}+3s+2}$, whose realization might involve a gain and a summation block, both of which are approximations. Second, by cascading subsystems using serial decomposition a system may be susceptible to error accumulation due to realization approximation. In contrast, parallel decomposition avoids the issue of error accumulation and amplification. Third, a strictly proper transfer function can always be decomposed as a summation of strictly proper transfer functions, which can be modeled by the three elementary modules described in Eq (5). Furthermore, we show that the three elementary modules allow exact CRN implementation using our configurable primitive components. For these reasons, parallel decomposition may be preferable to serial decomposition.

Nevertheless, applying parallel decomposition exclusively may not always be the best choice. The reasons are twofold. First, parallel decomposition requires one final summation block to sum up all elementary modules. This summation block, when it is implemented with a CRN, is approximative. Second, a hybrid decomposition strategy may achieve more effective implementation than applying a parallel decomposition. For instance, consider the system of transfer function $T=\frac{3s^{2}+8s+6}{\left(s+1)(s+2)(s^{2}+2s+2\right)}$. If only parallel decomposition is applied, then *T* is decomposed into $\frac{2}{s^{2}+2s+2}+\frac{1}{s+1}-\frac{1}{s+2}$. Nevertheless, if serial decomposition is also exploited, then $\frac{1}{s+1}-\frac{1}{s+2}$ can be rewritten as $\frac{1}{\left(s+1)(s+2\right)}$, which can be implemented by cascading two gain blocks with (*k*_0_, *k*_1_) = (1, 1) and (2, 1), respectively. Therefore, with this rewriting a final summation block only needs to sum up the outputs of the two subsystems rather than three subsystems. Furthermore, the hybrid parallel and serial decomposition strategy may be more cost effective than using parallel decomposition alone. Future work should exploit a good hybrid decomposition strategy and remove the approximative effect due to the final summation block in parallel decomposition.

### DSD Reaction Rates

In this paper, we realize all CRNs with DSD reactions. Typically, the rate constants in DSD reactions are approximately 10^−3^ nM^−1^ s^−1^ [33]. For example, if one requires the parameter $k_{0}=r_{0}^{\pm }z^{\pm }$ in the degradation reaction to be 100 s^−1^, then the concentrations of *z* ^±^ should be of magnitude 10^5^ in nM. However, such a high concentration might be impractical. To alleviate this high concentration requirement, one may try to increase the DSD reaction rates. Zhang *et al.* constructed and characterized DNA catalytic circuits driven by entropic gains [28]. Based on the entropy effects, a variant called the tethered entropy driven catalytic circuits was introduced [35, 38] to shorten the catalytic cycle and improve the reaction kinetics using localized hybridization reactions, which are achieved by tethering the key species in DSD reactions to increase their local concentrations. The influence of tethering is effectively presented in a speed-up factor *λ*, whose value can be feasibly reach approximately 10^5^ [35]. Thus, the toehold binding rate constants can be scaled up to *λ* × 5 × 10^−5^ nM^−1^ s^−1^, where 5 × 10^−5^ nM^−1^ s^−1^ is the short toehold binding rate constant in diffusion-based systems [6].

In our DSD simulation, we assume the factor *λ* = 1000 is available to increase the toehold binding rate constants to approximately 0.05 nM^−1^ s^−1^. Effectively, the concentrations of the auxiliary species can be reduced by a factor of 25. Under the toehold binding rate increase, a catalysis has a rate constant approximately 0.025 nM^−1^ s^−1^ in our interested range of concentrations under the intended system operation. Thus, the kinetics of the products *y* ^±^ will now become $\dot{y^{\pm }}=0.025x^{\pm }u^{\pm }$. If *x* ^±^ could be tuned to 1000 nM, then we can have an integration block with weight as high as 25.

Although we have reduced the required concentrations of auxiliary species using the speed-up factor *λ*, there are still some limitations. First, if *β* is set to 500 in the elementary modules $\frac{D_{2}}{s^{2}+\beta s+\gamma }$ and $\frac{D_{3}s}{s^{2}+\beta s+\gamma }$ shown in Fig 12, then we have to prepare the catalysts *z* ^±^ with large concentrations (approximately 500/0.05 = 10000 nM due to the relation $\beta =r_{0}^{+}z^{+}=r_{0}^{-}z^{-}$). In contrast, if $D_{3}=500=r_{1}^{+}x^{+}=r_{1}^{-}x^{-}$, then the concentrations of *x* ^±^ would be 500/0.025 = 20000 nM. Thus, although we have reduced the concentrations of the auxiliary species to increase the range of coefficients in the transfer functions, further reduction may be needed.

### Fuel Supply

DSD is a relatively mature technology for reliable *in vitro* construction of biochemical systems, and is more practical than *in vivo* experiments. However, one of the main obstacles for practical DSD experiment is to provide constant supply of fuels [37]. This paper assumes the fuels are large in quantity and ignore the situations where the fuels become deficient. In reality, the fuels are converted to inert waste and one has to continually provide the fuels to the system. Providing stable fuel supply can be challenging in a cellular context. Therefore, it is easier to implement DSD reactions *in vitro* instead of *in vivo*.
